# Long-term biogas slurry fertilization alters rhizosphere soil metabolite profiles and microbial communities in poplar plantations

**DOI:** 10.3389/fmicb.2025.1727035

**Published:** 2026-01-06

**Authors:** Xiao-Xiao Gao, Xing-Ye Yu, Chun-Zhi Jin, Long Jin, Su Bu, Taihua Li, Hong-Hua Ruan, Kee-Sun Shin, Feng-Jie Jin

**Affiliations:** 1 College of Ecology and Environment, Co-Innovation Center for Sustainable Forestry in Southern China, Nanjing Forestry University, Nanjing, China; 2 Korea Research Institute of Bioscience and Biotechnology (KRIBB), Daejeon, Republic of Korea; 3 College of Life Science, Nanjing Forestry University, Nanjing, China; 4 National Positioning Observation Station of Hung-tse Lake Wetland Ecosystem in Jiangsu Province, Hongze, China

**Keywords:** biogas slurry fertilization, metabolites, microbial community, poplar plantations, rhizosphere soil

## Abstract

Biogas slurry, a nutrient-rich organic fertilizer, has unclear impacts on plant-soil interactions. Rhizosphere metabolites serve as a bridge between plants and soil microorganisms, significantly influencing soil health and microbial activity. This study employed high-throughput sequencing and untargeted metabolomics to investigate microbial community and metabolite profiles in rhizosphere soil of poplar plantations treated with biogas slurry at different concentrations (Con: untreated; Low: 250 m^3^ ha^−1^ yr^−1^; High: 375 m^3^ ha^−1^ yr^−1^). Results showed that long-term application of biogas slurry significantly increased soil total nitrogen (TN) and available phosphorus (AP) levels, thereby enhancing soil fertility. Compared to fungi, biogas slurry treatment had a greater effect on bacterial community richness. Biogas slurry application also resulted in a significant decrease in organic acid content and a significant increase in nucleosides and nucleotides, saccharides, and esters in rhizosphere soil metabolites. Furthermore, differential metabolites between treatment groups were mainly classified into organic acids, organic bases, nucleosides and nucleotides, among others. Redundancy analysis (RDA) revealed that organic acids were positively correlated with NO_3_^−^, alkaline phosphatase (ALP) and pH, but negatively with TN and AP. KEGG pathway enrichment analysis revealed that in the Low vs. Con comparison group, differential metabolites were primarily enriched in amino acid metabolism, lipid metabolism, carbohydrate metabolism, and nucleic acid metabolism pathways. Co-occurrence networks indicated that the bacteria-soil properties-metabolites network was more complex than the fungal network. Our results suggest that low-concentration biogas slurry exerts stronger regulatory effect on rhizosphere metabolites and recruits beneficial microorganisms more effectively, with a lower ecological risk such as plant diseases.

## 1 Introduction

In forest field management, fertilization offers accessible nutrients and is the most commonly adopted and effective measure to stimulate plant growth. However, the long-term and large-scale application of chemical fertilizers will result in the accumulation of excessive minerals in the soil, accelerate the loss of soil organic carbon (SOC), reduce the resistance of plants to pests and diseases, and endanger soil health, thereby undermining ecosystem function (de França et al., 2021; Wang H. et al., 2024). Therefore, it is crucial to adopt a reasonable and effective fertilization method. Biogas slurry, an anaerobic digestate primarily derived from crop straw, animal manure, and human excreta (Insam et al., 2015). Biogas slurry contains inorganic salts such as sodium salts and phosphates, trace elements such as iron, manganese, and copper (Merlin Christy et al., 2014), and a variety of hydrolases such as cellulase and protease, which have positive effects on plant growth and soil environment (Mohanty et al., 2024; Tang et al., 2019). Several studies have demonstrated that the long-term application of biogas slurry increased soil organic carbon and environmental nutrients, promoted plant growth, and had great significance for the positive succession of the microbial community structure (Abubaker et al., 2013; Liu et al., 2024; Xu et al., 2019).

Healthy soil constitutes a harmonious and complex social system, where interactions among productive elements endow it with a favorable structure and optimal functionality. Bacteria and fungi are important participants in soil ecological processes and are closely related to the soil environment. Studies have indicated that long-term application of biogas slurry can directly alter soil physicochemical properties and improve soil quality. This is mainly attributable to the high levels of organic matter and mineral nutrients—nitrogen, phosphorus, and potassium—present in the biogas slurry (Rahaman et al., 2021; Yan et al., 2021). In agricultural soils, applying biogas slurry can alter soil pH, nitrate-nitrogen, quick-acting potassium, etc., and have a preventative effect on certain plant diseases (Du et al., 2018; Li N. et al., 2023; Li et al., 2024; Tang et al., 2021). However, excessive application of biogas slurry might cause toxic effects (Bolan et al., 2003), salinization, and soil nutrient loss (Godos et al., 2010). Soil microorganisms are a comprehensive biological indicator of soil quality. The application of biogas slurry can influence the soil microbial community by modifying soil properties, and may also have a direct impact (Terhoeven-Urselmans et al., 2009). The researchers indicated that the diversity and richness of soil bacterial and fungal communities increased significantly after the application of biogas slurry (Li M. et al., 2023; Sun et al., 2023). It should be noted that one study suggested that the application of biogas slurry had a limited impact on the α-diversity of soil bacterial and fungal communities, and reduced the correlation and aggregation of bacterial and fungal symbiotic networks (Liang et al., 2023). The disparity in the results might be attributed to the different types of biogas slurry and experimental subjects. The alteration in soil microbial community structure might be a comprehensive response to plant feedback and the soil environment, but the specific mechanism remains unclear. Therefore, this study will deeply explore the possible mechanism of the influence of biogas slurry on soil microorganisms.

The rhizosphere, a critical interface in soil ecology, facilitates complex interactions among microorganisms, plant roots, and soil components, driving biogeochemical processes. Rhizosphere metabolites are the links of mutual connections and interactions among the three and are predominantly composed of root exudates and microbial metabolites. Root exudates play a significant role in nourishing plant development and bolstering resistance to biotic stresses, such as pests and diseases, as well as regulating the beneficial rhizosphere microbiota (Li et al., 2025; Wang Q. et al., 2024; Zheng et al., 2024). Microbial metabolites actively contribute to improving soil quality and nutrient uptake by plants, modulating geochemical processes in the rhizosphere, and increasing plant resistance to biotic and abiotic stresses (Bölscher et al., 2024; Miura et al., 2020; Xue et al., 2024). Fertilization practices adjust soil properties and microbial communities, affecting the rhizosphere metabolome. In turn, metabolome variations induce adaptive responses in soil microbes and change soil characteristics. Studies have shown that rhizosphere metabolites are compounds (e.g., organic acids, sugars, amino acids, phenolics, fatty acids, etc.) produced through enzymatic reactions, which enrich soil organic carbon, alter soil physical and chemical properties, and release material signals for changes in soil microbial structure and function (Guerrieri et al., 2019; Koprivova and Kopriva, 2022; Zhao Y. et al., 2022). Certain organic acids and polysaccharides in root exudates facilitate microbial decomposition of soil organic matter (SOM) (Meier et al., 2017), but can also suppress it by controlling the structure of humic acids and the biomass of microbes (Sun et al., 2021). The relationship between microbial community composition and rhizosphere metabolites varies across the rhizosphere soils of different plant species (Che et al., 2024; Rempfert et al., 2024; Wang et al., 2023). Variations in soil quality, root activity, and microbial composition within the rhizosphere highly respond to alterations in rhizosphere metabolite profiles. Therefore, it is particularly essential to understand the rhizosphere soil state and plant-microbial interactions, and metabolomics can explain this relationship at the molecular level.

The objective of this study is to explore the responses of soil ecosystem components following the application of biogas slurry to evaluate its potential as a fertilizer in forestry. The plant root system acts as a principal mediator for the exchange of materials and information. Consequently, we investigate the rhizosphere soil of poplar plantations during their period of maximal fine root development in April. We analyzed differences in the rhizosphere soil physicochemical properties, microbial community structure, and metabolite profiles in poplar plantations treated with various concentrations of biogas slurry, to assess the integrated effects of biogas slurry application on the plant–soil-microbe system. We expect to address the following issues to provide solid evidence for green and efficient fertilizer application in forestry: (1) How does the application of biogas slurry affect soil fertility and microbial activity in the rhizosphere soil? (2) How do different treatments lead to variations in metabolite profiles in the rhizosphere soil? (3) What are the potential relationships and interaction mechanisms among soil physicochemical properties, microbial community structure, and metabolite composition following biogas slurry application? We hypothesize that biogas slurry application will significantly alter the physicochemical properties of rhizosphere soil, enhance microbial diversity and modify community structure, and induce distinct variations in metabolite profiles. These changes are anticipated to be concentration-dependent, with lower concentrations of biogas slurry potentially offering the most favorable outcomes for soil health and fertility.

## 2 Materials and methods

### 2.1 Experimental site and soil sampling

The experimental site was set up in a forest farm (32°52′N, 120°49′E) near the Yellow Sea Forest in Jiangsu Province, eastern China, with a total area of approximately 12.6 ha. Experimental treatment and unified management began in 2012 with a 10-year-old pure poplar (*Populus deltoids* cv. ‘I-35’). The forest farm belongs to the subtropical monsoon climate, with an average annual temperature of 13.7 °C and an annual precipitation of 1,051 nm. The soil is covered as a desalted sandy loam meadow with a slightly alkaline pH (~8) (Han et al., 2018). The initial physical and chemical characteristics of the soil in the experimental plot are detailed in Supplementary Table S1.

Random block design was adopted, including three replicated blocks, each plot was divided into three different treatments (20 × 20 m), the distance between plots was 500 m, and the poplar spacing was 5 × 5 m. The experimental plots were sprayed with quantitatively graded concentrations of biogas slurry in May, August, and October annually. The treatments were as follows: (1) an untreated control group (Con), (2) a low-concentration biogas slurry applied at a rate of 250 m^3^ ha^−1^ yr^−1^ (Low), and (3) a high-concentration biogas slurry applied at a rate of 375 m^3^ ha^−1^ yr^−1^ (High). The basic physicochemical properties of biogas slurry were measured and described in Supplementary Table S2. In April 2019, samples from three different treatment plots were collected on the rhizosphere margin soil (0–20 cm) of poplar plantation for subsequent data determination.

### 2.2 Determination of soil physicochemical properties

Rhizosphere soil samples from nine plots under the three treatments were randomly selected, mixed evenly, and then the soil samples obtained through a 2 mm sieve were used for soil physicochemical analysis. We used the previously described methods to determine soil pH, moisture content (MC), total carbon (TC), total nitrogen (TN), nitrate nitrogen (NO_3_–N), and available phosphorus (AP) (Ren et al., 2020). The microbial biomass carbon (MBC) and microbial biomass nitrogen (MBN) were determined via a chloroform (CHCl_3_)-0.5 M K_2_SO_4_ extraction method using an automated TOC analyzer (TOC-VCPH; Shimazu Inc., Japan) (Frey et al., 2004). The activity determination of alkaline phosphatase (ALP) and nitrate reductase (NR) was also based on previous studies (Schinner et al., 1996; Tabatabai, 1994).

### 2.3 Genomic DNA extraction, PCR amplification, and sequencing

Genomic DNA from rhizosphere soil of Poplar was extracted utilizing the FastDNA Spin Kit for Soil (MPbio, CA, USA), and the quality of the extracted DNA was assessed through 0.8% (w/v) agarose gel electrophoresis. The concentration and purity of the DNA were determined using a NanoDrop 2000C spectrophotometer (Thermo Fisher Scientific, MA, USA) (Zhao Z. et al., 2022). For bacterial diversity assessment, the target region was the 16S V3-V4 region (primers: 343F 5′-TACGGRAGGCAGCAG-3′; 798R 5′-AGGGTATCTAATCCT-3′), while for fungal diversity analysis, it focused on ITS2 region (primers: ITS3F: 5′-GCATCGATGAAGAACGCAGC-3′and ITS4R: 5′-TCCTCCGCTTATTGATATATGC-3′) (Fujita et al., 2001; Nossa et al., 2010). PCR amplification was conducted following the protocol established by Yu et al. (2022), after which the amplicons were purified and quantified before being sequenced on an Illumina Hiseq2500 platform.

Sequence assignment was performed using Mothur software (V1.35.1),^1^ retaining only valid clean tags, and subsequent sequence analysis was performed utilizing USEARCH software, with sequences exhibiting ≥97% similarity classified into an operational taxonomic unit (OTU) (Nguyen et al., 2016). SILVA (16S) and Unite (ITS) databases were employed for annotating the taxonomic data and normalizing OTU abundance data against standard sequence numbers. The raw sequencing data have been deposited in the National Center for Biotechnology Information (NCBI) Sequence Read Archive (SRA) under the Bioproject accession number PRJNA672251, with 16S rDNA sample accession numbers ranging from SRR31817989 to SRR31817997 and ITS sample accession numbers spanning from SRR31799612 to SRR31799620.

### 2.4 Determination of rhizosphere soil metabolites

A non-targeted metabolomics approach utilizing LC-QTOF-MS was employed to conduct a comprehensive metabolomics analysis of rhizosphere soil samples as follows: 0.3 g of soil sample (measured with an accuracy of 0.0001 g) was weighed, followed by the addition of 1 mL of a mixture comprising methanol, acetonitrile, and water in a ratio of 2:2:1. The mixture was vortexed for 30 s to ensure thorough mixing and then subjected to ultrasonic treatment for 10 min. Subsequently, centrifugation was performed at 4 °C for 15 min at a speed of 13,000 r/min; thereafter, 700 μL of the supernatant was collected and placed into a vacuum freeze-dryer until complete evaporation occurred. 100 μL of acetonitrile-water (1:1) solution was added to dissolve the mixture, followed by another round of vortexing for 30 s and ultrasonic treatment for an additional 10 min. A final centrifugation step at the same conditions yielded another supernatant from which we took out 50 μL to transfer into an injection vial for subsequent detection via LC-MS (Cheng et al., 2022). The untargeted metabolomics data have been deposited to MetaboLights repository with the study identifier MTBLS12235.

### 2.5 Statistical analysis

Data were processed and analyzed using Excel 2019, SPSS 27.0, and QIIME2, and plotted using Origin 2024 and R. Shannon diversity index and richness metrics were computed using QIIME2 (2021.04), while the alpha diversity of the soil microbial community between treatments was plotted via Origin 2024. Pearson’s correlation coefficient was used to assess the correlation between physicochemical properties and soil microbial diversity. One-way analysis of variance (ANOVA) alongside least significant difference (LSD) tests were used to evaluate differences in physicochemical parameters, relative abundance of microbial community phyla and genera, as well as relative content of metabolites between treatments. The relationship between rhizosphere microbial community composition (OTU abundance) and environmental factors was examined through redundancy analysis (RDA). Metabolites underwent annotation and analysis via the KEGG database, with results from differential metabolite screening represented visually in the form of a volcano plot. Co-occurrence networks based on Spearman correlations among soil properties, microbial communities, and metabolites were analyzed using the igraph package within R software, subsequently visualized using ChiPlot.^2^ Data for each treatment are presented as means accompanied by standard errors; differences between treatments are expressed as *p* < 0.05. Structural equation modeling (SEM) was used to test the hypothesized causal relationships among biogas slurry treatment, rhizosphere soil metabolites, microbial diversity, microbial community structure, and soil properties. Model construction and validation followed the methods described in previous research (Zhang et al., 2026), and all analyses were conducted in R using the lavaan package (Rosseel, 2012).

## 3 Results

### 3.1 Physicochemical properties of poplar rhizosphere soil

The long-term application of biogas slurry was observed to significantly enhance the available phosphorus (AP) content in rhizosphere soil in comparison with the control group. Notably, the application of a low concentration of biogas slurry resulted in a substantial increase in TN levels while simultaneously reducing the C/N ratio in rhizosphere soil. In addition, microbial biomass carbon (MBC) content was significantly diminished due to high-concentration biogas slurry treatment (Table 1). Beyond these, no significant impacts were discerned on other soil physicochemical properties, including moisture content (MC), total carbon (TC), nitrate nitrogen (NO_3_^−^–N), etc.

**Table 1:** Effects of biogas slurry application on physical, chemical properties and enzyme activities of poplar rhizosphere soil.

Attribute	Con	Low	High
Moisture content	0.21 ± 0.00^a^	0.21 ± 0.01^a^	0.20 ± 0.01^a^
pH	7.65 ± 0.02^a^	7.63 ± 0.02^a^	7.67 ± 0.03^a^
Total C (g kg^−1^)	4.71 ± 0.05^a^	5.06 ± 0.30^a^	5.10 ± 0.21^a^
Total N (mg kg^−1^)	317.33 ± 4.83^b^	401.75 ± 28.25^a^	350.45 ± 6.25^a,b^
C/N	14.83 ± 0.08^a^	12.61 ± 0.14^b^	14.57 ± 0.70^a^
Nitrate (mg kg^−1^)	38.01 ± 5.12^a^	41.55 ± 7.22^a^	49.86 ± 1.95^a^
Available P (mg kg^−1^)	0.61 ± 0.04^b^	1.07 ± 0.05^a^	1.22 ± 0.13^a^
Microbial biomass C (mg kg^−1^)	793.56 ± 15.45^a^	795.72 ± 28.42^a^	695.72 ± 20.47^b^
Microbial biomass N (mg kg^−1^)	8.70 ± 1.72^a^	18.74 ± 1.55^a^	12.10 ± 3.48^a^
Alkaline phosphatase (mg kg^−1^ 24 h)	1.25 ± 0.08^a^	1.31 ± 0.02^a^	1.46 ± 0.10^a^
Nitrate reductase (μmol/d/g)	2.61 ± 0.05^a^	2.72 ± 0.08^a^	2.64 ± 0.06^a^

The values were presented as mean ± standard error (*n* = 3), with different lowercase letters indicating significant differences (*p* < 0.05) between treatments. Con-no treatment control; Low-biogas slurry (250 m^3^ ha^−1^ yr^−1^) treatment; High-biogas slurry (375 m^3^ ha^−1^ yr^−1^) treatment.

### 3.2 Effects of biogas slurry application on the rhizosphere soil microbial community in poplar plantations

**Figure 1:**
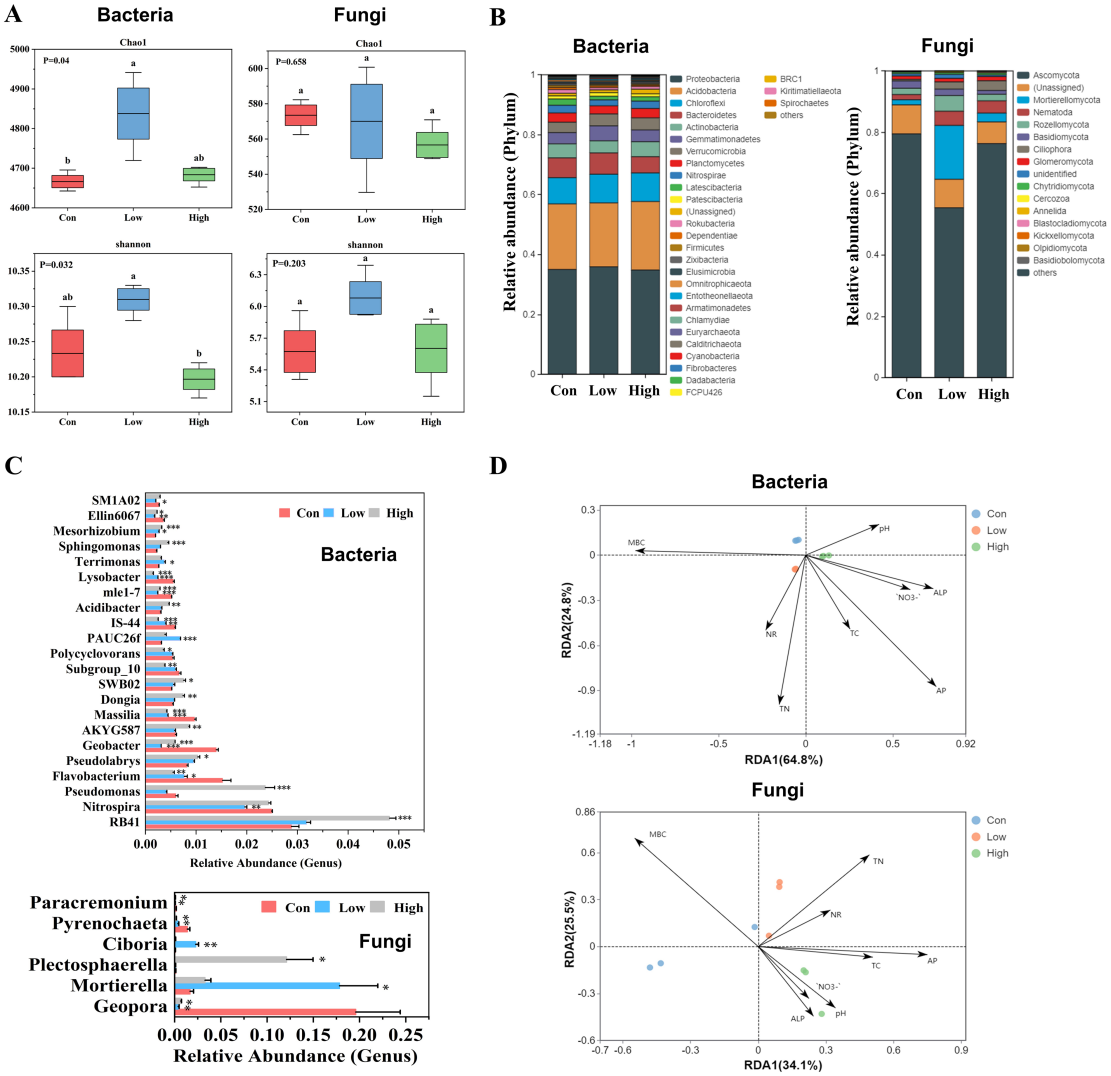
Impact of biogas slurry addition on rhizosphere soil microbial communities. **(A)** Richness and Shannon index of bacterial and fungal communities in rhizosphere soils. **(B)** Bacterial and fungal community compositions of rhizosphere soil at the phylum level (Top 30). **(C)** Comparison of the relative abundances of the Top 30 microorganisms with significant differences among different treatments at the genus level. The error bars represent the standard errors of three repetitions. *, **, and *** indicate that there were significant differences at *p* < 0.05, *p* < 0.01, and *p* < 0.001, respectively, between the biogas slurry treatment and the control group. **(D)** Redundancy analysis (RDA) between environmental factors and the rhizosphere soil microbial communities. MBC, microbial biomass carbon; TC, total carbon; TN, total nitrogen; NO_3_^−^, nitrate-nitrogen; AP, available phosphorus; ALP, alkaline phosphatase; NR, nitrate reductase. Con-no treatment control; Low-biogas slurry (250 m^3^ ha^−1^ yr^−1^) treatment; High-biogas slurry (375 m^3^ ha^−1^ yr^−1^) treatment. Different lowercase letters indicated a statistically significant difference between treatments (*p* < 0.05) (*n* = 3).

Alpha diversity analysis indicated that application of low-concentration biogas slurry significantly increased the richness of the rhizosphere soil bacterial community, but had no significant effect on the bacterial Shannon diversity (Figure 1A). In contrast, compared with the control group, treatments with either low or high concentrations of biogas slurry exerted no significant influence on Chao1 and Shannon indices of fungal community. Venn diagram analysis revealed that the biogas slurry application substantially increased bacterial sequence reads in the order of High > Low > Con (Supplementary Figure S1), whereas its effect on the fungal community was negligible.

The composition and relative abundance of rhizosphere soil microbial communities were further investigated (Figures 1B,C), focusing on the top 30 most abundant taxa at phylum and genus levels. At the phylum level (Figure 1B), the dominant bacterial communities in this poplar plantation before and after long-term biogas slurry application were identified as *Proteobacteria* and *Acidobacteriota*, among others. The application of biogas slurry significantly enhanced the abundance of *Chloroflexi* and *Verrucomicrobia*, but decreased that of Planctomycetes, *Nitrospirae* and *Latescibacteria*. Applying high concentration of biogas slurry also significantly reduced the abundance of Bacteroidetes. In terms of the composition of the fungal community, the application of low-concentration biogas slurry significantly increased *Mortierellomycota* and *Rozellomycota*, and significantly reduced the most dominant fungi *Ascomycota* and *Cercozoa* (Figure 1B). At the genus level (Figure 1C; Supplementary Tables S3, S4), the majority of bacterial communities exhibited a significant response to biogas slurry treatment. Specifically, the application of biogas slurry notably increased the abundance of *Mesorhizobium* and decreased that of *Nitrospira*, *Flavobacterium*, and *Geobacter*. With increasing biogas concentration, the abundance of *RB41*, *Pseudomonas*, and *Pseudolabrys* significantly rose. In addition, the relative abundance of the fungal genus *Geopora*, *Pyrenochaeta*, and *Paracremonium* was significantly reduced by biogas slurry application, while the application of a low concentration of biogas slurry significantly increased the abundance of *Mortierella* and *Ciboria*. It is noteworthy that the application of a high concentration of biogas slurry significantly increased *Plectosphaerella*. Overall, the effect of low concentrations of biogas slurry on the fungal community was greater than that of high concentrations. RDA analysis showed that fungal communities treated with different biogas slurry concentrations along RDA axes 1 and 2 were significantly better than those of the bacteria (Figure 1D). We further explored the correlation between microbial diversity and soil properties, and the results showed that fungal community was positively correlated with TN and AP, and negatively correlated with MBC. Other soil properties have no significant correlation with the fungal communities (Figure 1D).

### 3.3 Statistical analysis of metabolites in the rhizosphere soil of poplar plantations

**Figure 2:**
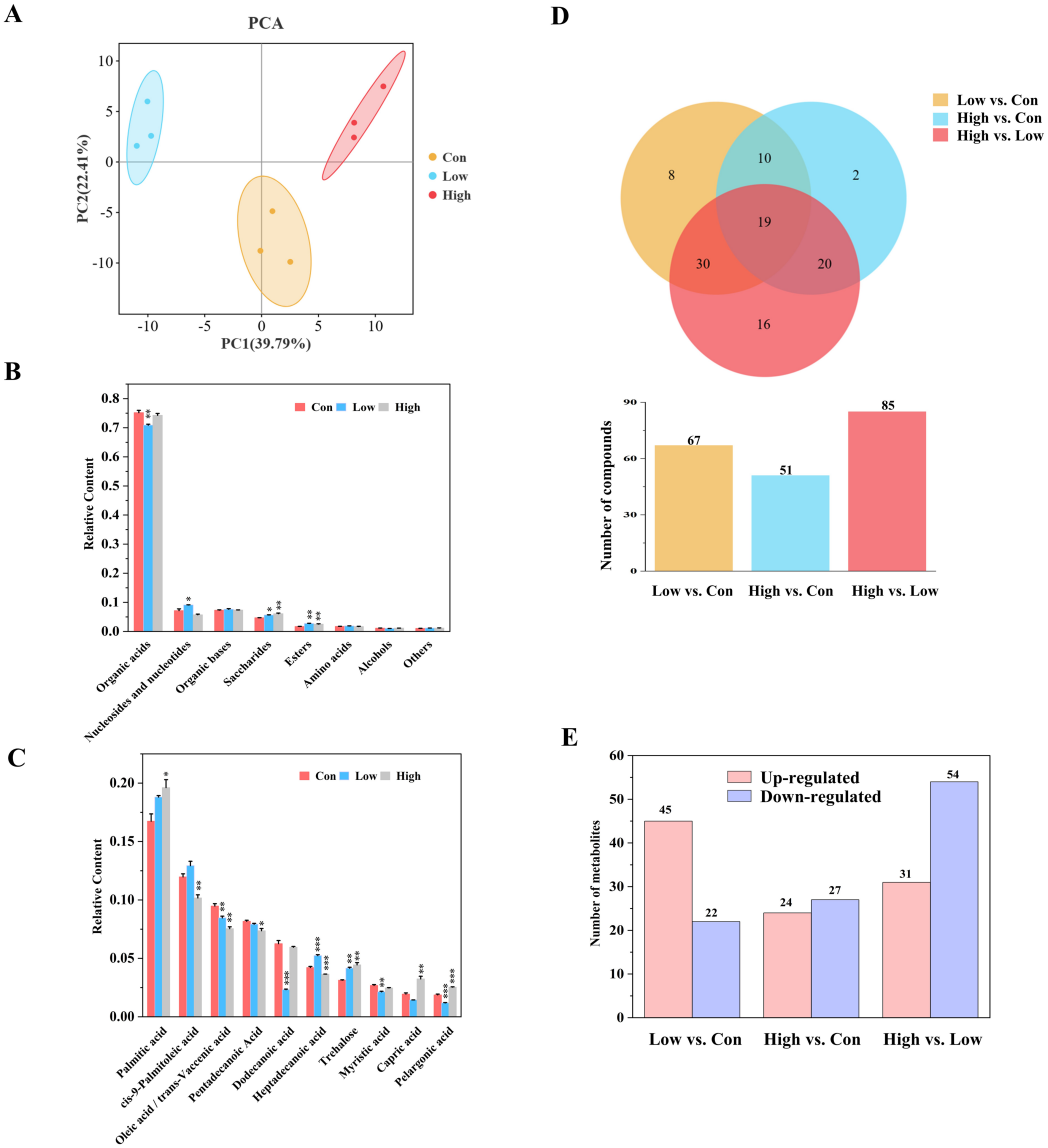
Metabolomic analysis among different treatments. **(A)** Principal component analysis (PCA) among different treatment groups. **(B)** Relative contents of different metabolite categories in rhizosphere soil treated with three concentrations of biogas slurry. **(C)** Relative contents of the major metabolites in rhizosphere soil treated with three concentrations of biogas slurry (top 10). The error bar represents the standard errors of three repetitions; *, **, and *** represent *p* < 0.05, *p* < 0.01, and *p* < 0.001, respectively, indicating statistically significant difference between the biogas slurry treatment and control group. **(D)** Venn diagram analysis of differential metabolites between treatment groups. **(E)** Numbers of up-regulated and down-regulated differential metabolites between different treatment groups.

The effect of biogas slurry at various concentrations on rhizosphere soil metabolites was further investigated. A total of 179 metabolites were identified from the poplar rhizosphere soil. Metabolite composition was significantly affected by the concentration of biogas slurry fertilization, and they were well distinguished based on PCA analysis (Figure 2A). These metabolites were primarily categorized into organic acids, nucleosides and nucleotides, organic bases, saccharides, esters, amino acids, alcohols, and so on. Among these, organic acids were predominantly dominant, constituting over 70% of the total metabolite content (Figure 2B; Supplementary Table S5). The main metabolites include long-chain saturated/unsaturated fatty acids such as palmitic acid, cis-9-palmitoleic acid, etc. (Figure 2C). The secretion of these metabolites was significantly influenced by the application of biogas slurry. Long-term treatment with biogas slurry inhibited the secretion of unsaturated fatty acids, especially cis-9-palmitoleic acid and oleic acid, while enhancing the secretion of saccharides, such as trehalose (Supplementary Table S5). Different concentrations of biogas slurry had varying effects on the secretion of saturated fatty acids. Venn diagram analysis revealed a higher number of differential metabolites in the Low vs. Con comparison group (Figure 2D). In comparison with the control, long-term treatment with low-concentrations biogas slurry induced changes in the levels of 67 metabolites, with 45 being up-regulated and 22 down-regulated (Figure 2E). In contrast, treatment with high-concentrations biogas slurry resulted in alterations of only 51 metabolites, of which 24 up-regulated and 27 down-regulated (Figure 2E; Supplementary Figure S2).

### 3.4 Differential metabolites

**Figure 3:**
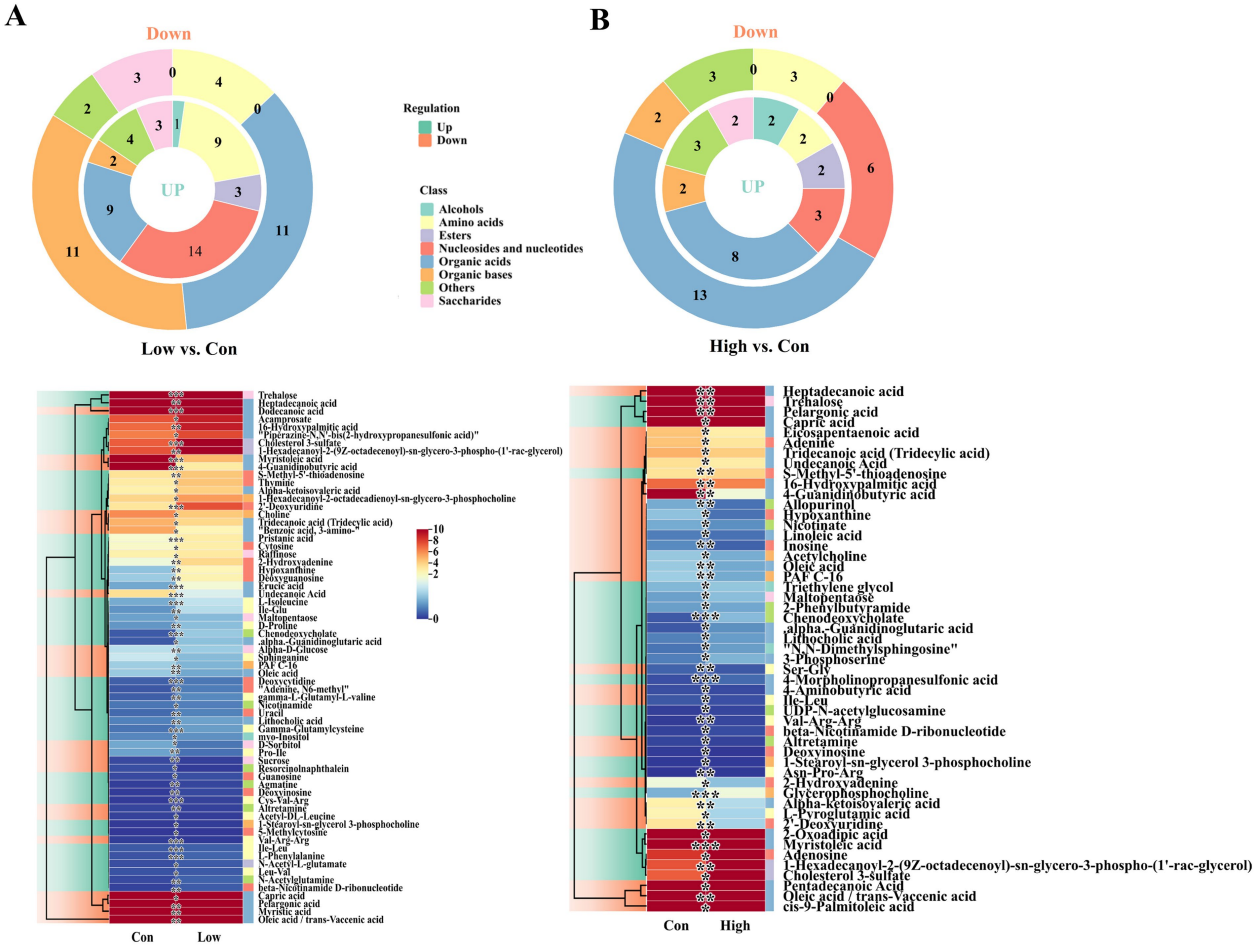
Classification statistics and expression pattern analysis of differential metabolites in rhizosphere soil between different treatments: **(A)** Low vs. Con; **(B)** High vs. Con.

The differential metabolites of rhizosphere soil between different treatment groups were further classified and statistically analyzed (Figure 3). Cluster analysis of differential metabolites demonstrated that the replicated samples subjected to different concentrations were distinctly separated in the heatmap (Supplementary Figure S3). Simultaneously, diverse concentrations of biogas slurry treatment demonstrated varying degrees of inhibition and promotion effects on metabolites in the rhizosphere soil (Supplementary Figure S3). In the Low vs. Con comparison group, the main differential metabolites were classified into organic acids (9 upregulated; 11 downregulated), nucleosides and nucleotides (14 upregulated), organic bases (2 upregulated; 11 downregulated), and amino acids (9 upregulated; 4 downregulated) (Figure 3A; Supplementary Table S6). Among these, the number of metabolites belonging to organic acids was close to that of the High vs. Con comparison group, while the quantities of the other three categories were much higher than those in the High vs. Con comparison group (Figure 3B; Supplementary Table S7). Furthermore, the count of differential metabolites categorized as saccharides in the High vs. Con comparison group was also notably lower than that in the Low vs. Con comparison group. Interestingly, low-concentration biogas slurry treatment led to a significantly higher number of upregulated metabolites in the categories of nucleosides and nucleotides and amino acids (14 and 9, respectively) compared to high-concentration biogas slurry treatment (3 and 2, respectively), while high-concentration biogas slurry application resulted in a greater number of downregulated nucleosides and nucleotides metabolites. Conversely, the Low vs. Con comparison group exhibited a higher number of down-regulated differential metabolites classified as organic bases (11 vs. 2). Collectively, these results indicated that rhizosphere soil metabolites did not increase proportionally with elevated biogas slurry concentration. Instead, the low-concentration treatment induced a greater abundance of up-regulated metabolites (Figure 3).

**Figure 4:**
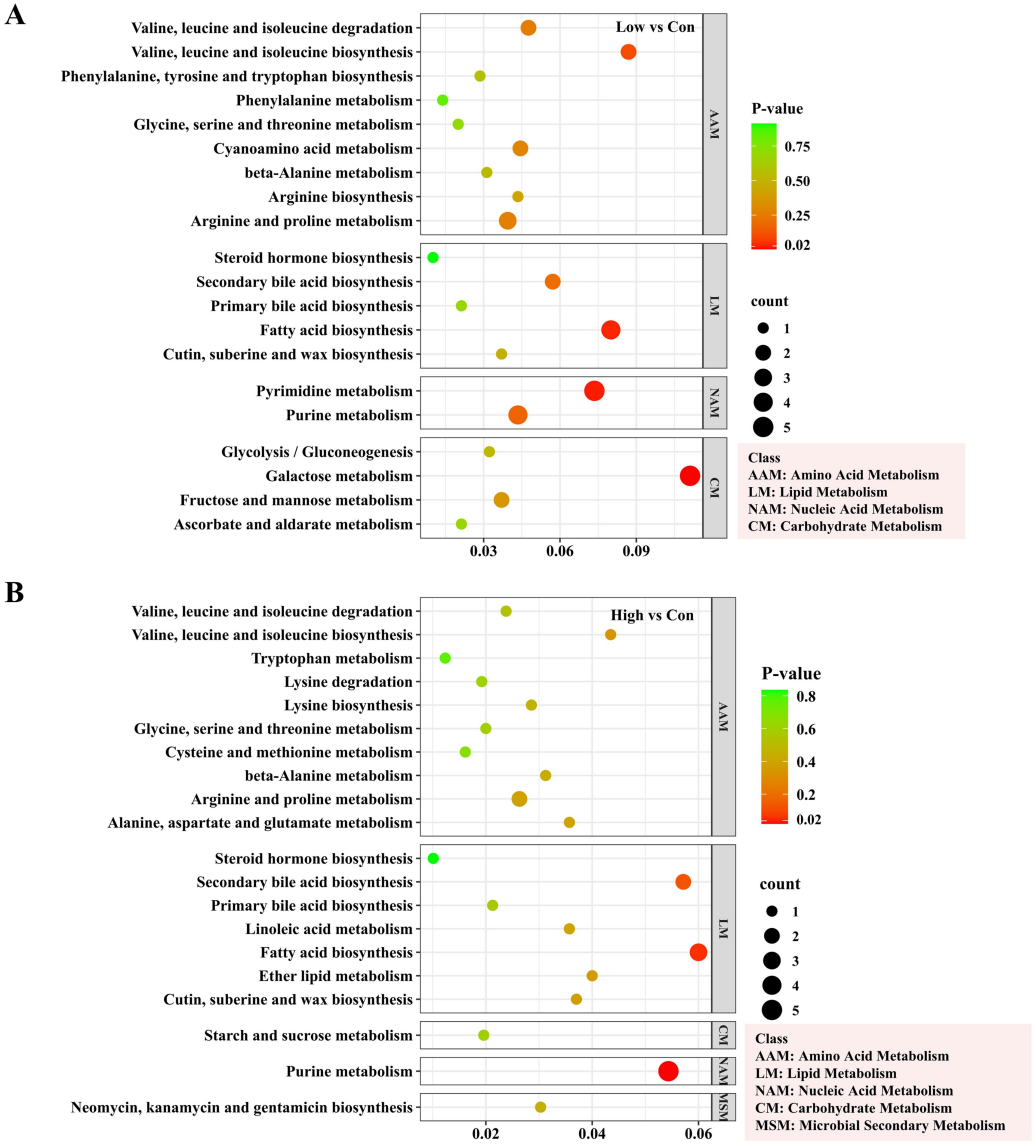
KEGG pathway enrichment analysis of differential metabolites between treatment groups (Top 20). **(A)** Low vs. Con; **(B)** High vs. Con.

KEGG pathway enrichment analysis showed that in the Low vs. Con comparison group, the differential metabolites were mainly enriched in the amino acid metabolism, lipid metabolism, carbohydrate metabolism and nucleic acid metabolism pathways, and were further subdivided and enriched significantly in the pyrimidine metabolism, galactose metabolism, and fatty acid biosynthesis pathways (Figure 4A; Supplementary Table S8). Within the pyrimidine metabolism pathway, metabolites such as uracil, cytosine, and deoxycytidine exhibited a significant upregulation, whereas in the fatty acid biosynthesis pathway, metabolites such as decanoic acid, dodecanoic acid, tetradecanoic acid, and octadecenoic acid were significantly downregulated. In contrast, the enrichment of the KEGG pathway for differential metabolites in the High vs. Con comparison group was not as significant as in the former, and was primarily enriched in the purine metabolism and fatty acid biosynthesis pathways (Figure 4B).

### 3.5 Relationships among rhizosphere soil properties, microbial communities, and metabolites

**Figure 5:**
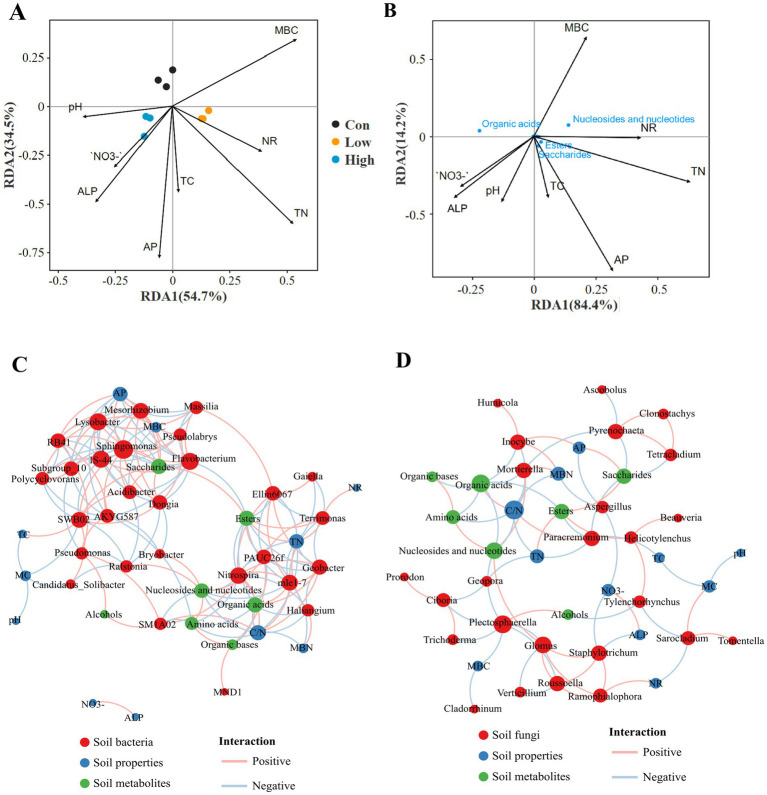
Correlation analysis. **(A)** RDA analysis between environmental factors and rhizosphere soil metabolites across varying biogas slurry concentrations. **(B)** RDA ordination evaluating associations between environmental factors and metabolite categories. MBC, microbial biomass carbon; TC, total carbon; TN, total nitrogen; NO_3_^−^, nitrate-nitrogen; AP, available phosphorus; ALP, alkaline phosphatase; NR, nitrate reductase. Co-occurrence network analysis among microbial community (**C**: bacteria; **D**: fungi), soil properties, and metabolites. The size of the nodes is scaled by the node degree. Links represent correlations between nodes (|*r*| > 0.7, *p* < 0.05). The pink and blue lines indicate positive and negative correlations, respectively.

Redundancy analysis (RDA) (Figure 5A) was used to assess the effects of varying biogas slurry concentrations on the relationship between rhizosphere soil environmental factors and metabolite composition. The results indicated that the rhizosphere soil metabolites treated with different biogas slurry concentrations could be effectively distinguished, and AP and TN mainly affect the composition of metabolites. The correlation between major rhizosphere metabolite categories and soil factors is depicted in Figure 5B, organic acids are positively correlated with NO_3_^−^, ALP, but negatively with TN, AP, NR, and MBC. Conversely, nucleosides and nucleotides exhibit an opposite trend.

Co-occurrence networks revealed significant correlations among the top 30 bacterial and fungal genera, physicochemical properties, and metabolites in rhizosphere soil. The bacteria-rhizosphere soil physicochemical properties-metabolites network (27 nodes, 49 edges) (Figure 5C) was more complex than the fungal network (25 nodes, 31 edges) (Figure 5D). The key environmental factors included the C/N ratio, TN, and AP; the main related bacterial genera were *Nitrospira* and *Flavobacterium*, and the main fungal genera were *Mortierella* and *Plectosphaerella*; the key metabolites in the rhizosphere soil were primarily organic acids, nucleotides, and esters. *Nitrospira* was associated with most rhizosphere soil metabolites, but only positively with organic acids. The C/N ratio was highly significant and positively correlated with *Geobacter*, but negatively correlated with *Haliangium*. Amino acids were negatively correlated with *AKYG587* and C/N ratio, and esters were positively correlated with TN and negatively correlated with *Flavobacterium* and *Geobacter*. Saccharides were negatively correlated with *Flavobacterium* and positively correlated with AP. In the fungal network, organic acids were negatively correlated with *Mortierella*, and nucleotides were negatively correlated with *Plectosphaerella* and positively correlated with *Mortierella*. Esters were positively correlated with *Helicotylenchus*, *Staphylotrichum* was negatively correlated with NO_3_^−^ and ALP, and *Sarocladium* was negatively correlated with NR.

**Figure 6:**
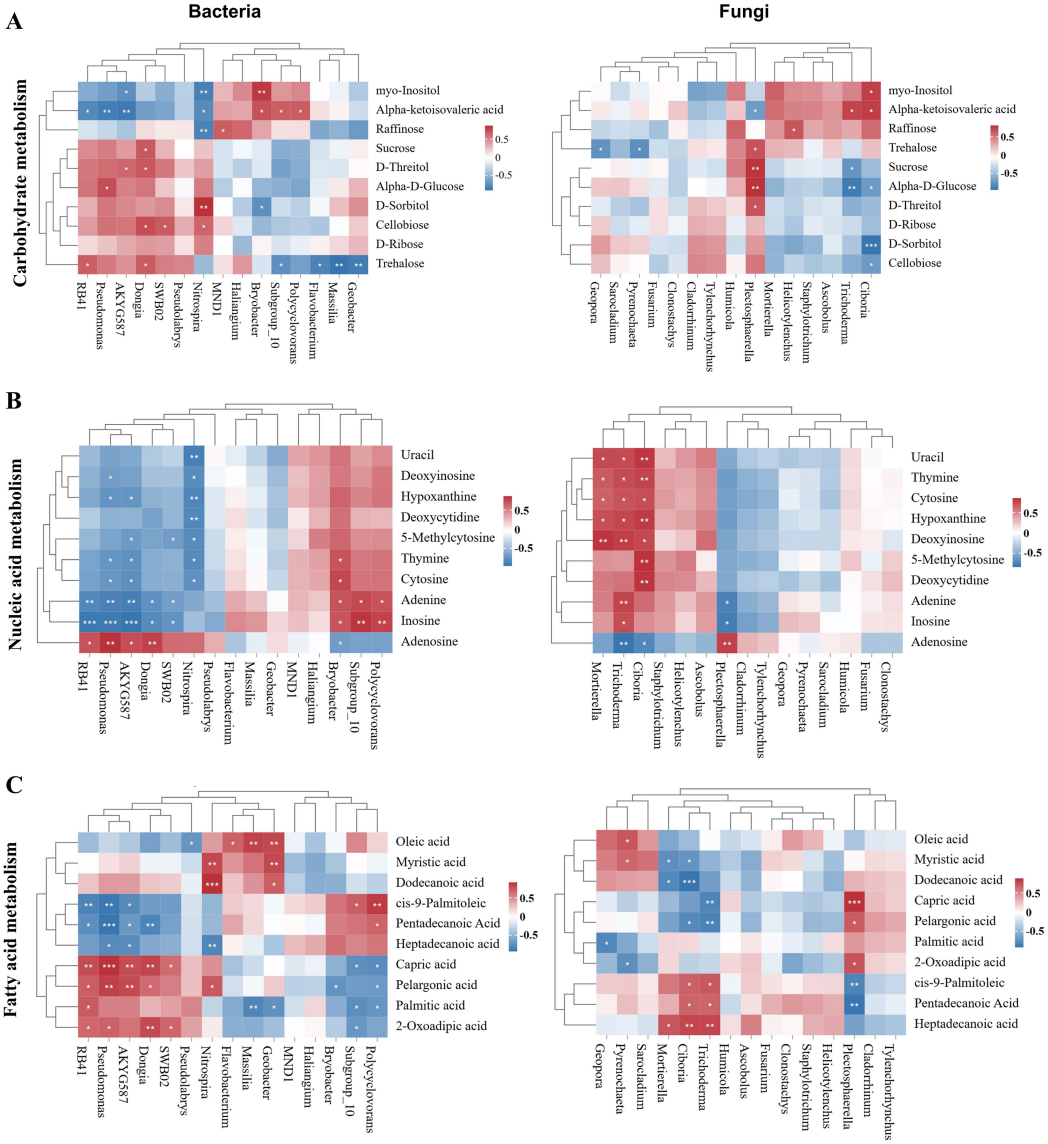
Heatmap analysis of the correlation between key differential metabolites and dominant microbial genera: **(A)** carbohydrate metabolism-associated metabolites vs. top 15 microbial (bacterial or fungal) genera, **(B)** nucleic acid metabolism-associated metabolites vs. top 15 microbial genera, **(C)** fatty acid metabolism-associated metabolites vs. top 15 microbial genera.

Based on the main metabolic pathways that the differential metabolites are involved in, the correlations between the major differential metabolites involved in carbohydrate metabolism, nucleotide metabolism, and fatty acid metabolism and the dominant microbial genera were further described (Figure 6). The main differential metabolites associated with carbohydrate metabolism, such as Trehalose, showed a significant negative correlation with bacterial genera *Geobacter*, *Massilia*, and *Flavobacterium*, and a positive correlation with *RB41* and *Dongia*. Additionally, bacterial genus *Nitrospira* and fungal genera *Plectosphaerella* and *Ciboria* were significantly correlated with most carbohydrate metabolism-related metabolites (Figure 6A). Interestingly, most of the key difference metabolites in the nucleotide metabolic pathway showed a significant negative correlation with bacterial genera such as *Pseudomonas*, *AKYG587*, and *Nitrospira*, while they were significantly positively correlated with fungal genera including *Mortierella*, *Trichoderma*, and *Ciboria*, for instance, Hypoxanthine, Thymine, Cytosine, etc. (Figure 6B). The main differential metabolites belonging to fatty acid metabolism, which were also the predominant metabolites extracted from the poplar rhizosphere soil, showed a more significant correlation with dominant bacterial genera. Specifically, cis-9-Palmitoleic acid, Pentadecanoic acid, Pelargonic acid, and Capric acid were significantly correlated with bacterial genera *RB41*, *Pseudomonas*, *AKYG587*, and *Polycyclovorans*, respectively, while the first three metabolites were also significantly related to fungal genera *Ciboria*, *Trichoderma*, and *Plectosphaerella* (Figure 6C).

**Figure 7:**
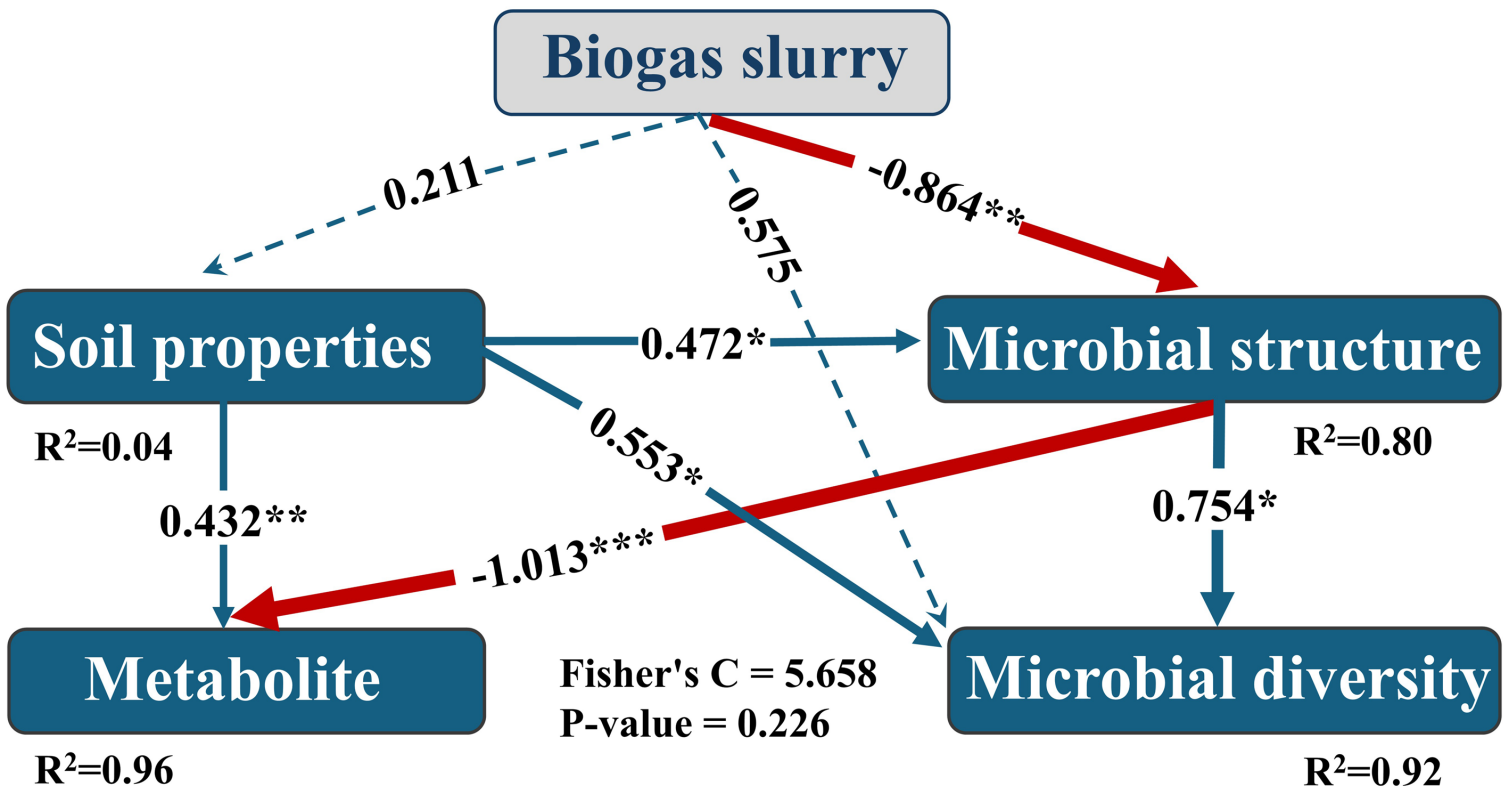
Structural equation modeling (SEM) analysis of rhizosphere physicochemical–microbial–metabolic interactions across biogas slurry application rates. Blue arrows indicate significantly positive effects, red arrows indicate significantly negative effects, and blue dotted arrows indicate non-significant effects at the 0.05 level. The standardized path coefficients for significant relationships are shown and are proportional to the arrow thickness. *p < 0.05, **p < 0.01, ***p < 0.001.

Structural equation model path analysis revealed that elevated biogas-slurry treatment had no significant effect on soil physicochemical properties, but markedly suppressed the rhizosphere soil microbial community (*p* < 0.01). Soil physicochemical traits positively influenced both the microbial community and its diversity (*p* < 0.05). Microbial community enhanced microbial diversity (*p* < 0.05) yet exerted a strong negative impact on soil metabolic function (*p* < 0.001). Furthermore, the direct effect of biogas-slurry treatment on microbial diversity was marginally significant (*p* = 0.093). Overall, these results demonstrate that biogas-slurry treatment can modulate rhizosphere microbial community structure via both direct and indirect pathways, thereby altering rhizosphere metabolite profiles (Figure 7).

## 4 Discussion

The utilization of biogas slurry enhanced the content of nitrogen (N) and phosphorus (P) related nutrients in the rhizosphere soil, reduced the rhizosphere soil C/N, and improved soil fertility (Table 1). Soil C/N indicates the N-use efficiency of the soil, and low C/N implies an increase in the effective N level in the soil surface layer (Mooshammer et al., 2014). This was partially attributed to the high NPK content of the biogas slurry and partially to the accumulation of microorganisms in the soil. However, the high-concentration biogas slurry treatment significantly reduced the content of MBC, which is distinct from some studies (Feng et al., 2024). This might be because some kind of heavy metals, antibiotics, etc., which are detrimental to microorganisms, could be present in the soil when applying a high concentration of biogas (Lu et al., 2021).

Soil microorganisms play a crucial role in elemental biogeochemical cycling, material turnover, and plant growth (Mohanram and Kumar, 2019). This study revealed that the application of biogas slurry notably enhanced the bacterial richness in the rhizosphere soil, but exerted no significant impact on fungi (Figures 1A,B). These findings are consistent with those reported by Cao et al. (2024). Previous research has suggested that rhizosphere microorganism diversity exhibits a specific response to plant growth stages (Bello et al., 2023), and plants secrete different compounds to adjust their microbial communities according to the season (Yao et al., 2017), which explains why, in our previous study, the fungal diversity was significantly higher after the application of biogas slurry, but no significant change was observed in bacteria (Yu et al., 2022). The disparate responses of fungi and bacteria to biogas slurry application could be attributed to the bacteria’s capacity to rapidly utilize available nutrients in the soil, whereas fungi are more proficient in decomposing complex organic matter, which may confer them a competitive advantage over fungi.

The application of biogas slurry exerted a more significant influence on the structure of the rhizosphere soil bacterial community than on the fungal one (Figures 1B,C). There was a notable increase in the abundance of bacteria belonging to the phylum *Acidobacteriota* and *Chloroflexi*, which are involved in the cycling of nutrients such as C and N in the soil. *Chloroflexi* is abundant in the soil and is conducive to promoting the oxidation of nitrite (Narsing Rao et al., 2022; Wu et al., 2024), while *RB41* plays a role in the material cycle and is capable of regulating plant performance and promoting plant growth (King and Weber, 2007). *Nitrospirae* oxidizes ammonia nitrogen in the digestate to nitrate (NO_3_^−^) for plant utilization; however, its abundance was significantly reduced. This might be attributed to a competitive relationship between *Nitrospirae* and *Chlorofiexi*, as well as the fact that *Chlorofiexi* is more adapted to anaerobic ammonia-rich environments (Bovio-Winkler et al., 2023). It is pertinent to note that the abundance of Bacteroidetes and *Gemmatimonadetes*, which are involved in the decomposition of organic matter, decreased significantly. The reason for this change could be that the biogas slurry contains rich available nutrients, which promote the growth of microorganisms that utilize these nutrients. Meanwhile, the bacteria involved in the decomposition of complex organic matter are no longer required to initiate their decomposition processes, resulting in a reduction in their overall activity and suppressed growth. A similar trend was observed in the fungal community, where applying a low concentration of biogas slurry significantly increased the plant growth-promoting rhizosphere fungus *Mortierella* and significantly decreased *Geopora*. Previous studies have demonstrated that *Mortierella* is capable of dissolving and releasing available P and K, promoting nutrient cycling for plant growth (Li et al., 2020; Ning et al., 2020), maintaining soil health, and inhibiting the growth of pathogenic bacteria (Wang et al., 2022). *Geopora* is an ectomycorrhizal fungus that has been demonstrated to promote soil C storage and C sequestration (Long et al., 2016). It is evident that the abundant available nutrients in biogas slurry restricted the growth of ectomycorrhizal fungi.

The use of biogas slurry has been recognized as a potential source of heavy metal and antibiotic contamination (Guo et al., 2018), and our findings also suggested favorable alterations in the abundance of specific environmental indicator microorganisms, particularly a remarkable increase in the abundance of *Sphingomonas* (Figure 1C). This bacterium is capable of degrading a diverse range of organometallic compounds, which may have beneficial impacts on environmental remediation (Asaf et al., 2020). *Geobacter* species generate energy by reducing iron oxides, but heavy metals in biogas slurry compete with Fe^3+^ for reduction sites, potentially damaging the structure and function of these bacteria, leading to a reduction in their abundance (Reguera and Kashefi, 2019). Several studies have shown that biogas slurry application can inhibit soil-borne diseases by modifying soil physicochemical and microbial properties (Cao et al., 2016; Hernández-Lara et al., 2022). *Pseudomonas* and *Pseudomlabrys* were significantly increased when applied at high concentrations, and some of their species could control pathogens by producing antibiotics and inducing systemic resistance (Venturi, 2022). It is noteworthy that the abundance of *Plectosphaerella*, a common plant-pathogenic fungus, increased by approximately 84-fold with the application of high-concentration biogas slurry (Figure 1C; Supplementary Table S4). In summary, the use of low-concentration biogas slurry led to a notable increase in the abundance of microorganisms involved in relevant nutrient cycling and resistance to pathogenic microorganisms, while phytopathogenic microbes rose dramatically with the application of high-concentration slurry. This may be due to the nutrient enrichment caused by high-concentration slurry application, which poses environmental risks.

The application of biogas slurry increased the levels of saccharides, nucleosides and nucleotide substances in the rhizosphere soil, while low-concentration treatment significantly reduced the relative content of organic acids (Figure 2B). Studies have shown that organic acids can provide abundant nutrients for microbial growth, enhance plant resistance, and have a role in the remediation of heavy metal pollution (Farhad et al., 2023; Guo-hui, 2012). The decrease in organic acid content might be attributed to the rich nutrients provided by biogas slurry application. Consequently, the rhizosphere may no longer need to secrete a large quantity of organic acids to maintain essential soil nutrient levels. However, the application of high-concentration biogas slurry did not result in a significant reduction in organic acid content, which may be due to heavy metal accumulation caused by biogas, stimulating plants and microbes to secrete certain organic acids cope with heavy metal stress. The decline in organic acid content also indicates that the intermediates of the TCA cycle are being consumed at an accelerated rate, indicating a shift in energy metabolism from a “maintenance state” to a “growth state.” The increase in nucleotide and nucleoside substances suggests an accumulation of raw materials for DNA/RNA synthesis, indicating active microbial replication and enhanced potential for community expansion. Among the main rhizosphere soil metabolites, in addition to the vast majority of organic acids, there is also a saccharide substance, trehalose (Table 1), whose increased abundance may be attributed to changes in soil nutrients and water content in the rhizosphere. Trehalose is widely distributed in plants, animals, and microorganisms and can assist organisms to resisting drought and other adverse stresses (Akram et al., 2015). In addition, biogas slurry application significantly increased the relative content of palmitic acid in the rhizosphere, which can enhance plant resistance to wilt pathogens (Luo et al., 2022). The relative contents of two unsaturated fatty acids (cis-9-palmitoleic acid and oleic acid), which can improve soil structure and soil quality, were significantly reduced (Yang et al., 2020), suggesting that biogas slurry application may have a positive impact on regulating soil physical properties. Interestingly, in our study, the application of biogas slurry at low concentrations led to an increase in the abundance of more metabolites (Figure 2E). KEGG pathway enrichment analysis of differential metabolites also indicated that the application of biogas slurry promoted nucleic acid (pyrimidine and purine metabolism) metabolic processes in rhizosphere soil, while inhibiting the biosynthesis of fatty acids (Figure 4). This aligns with our earlier microbial results, indicating that the influence of biogas slurry on the poplar rhizosphere micro-environment is concentration-dependent. Low-concentration amendments more effectively modulated soil microbial composition, enhanced rhizosphere metabolic activity, and prevented nutrient oversupply and pathogen risks associated with high-concentration treatments. These results provide valuable guidance for sustainable forestry-soil management.

Plants exert an influence on microorganisms by releasing bioactive molecules, which also have positive effects on plant growth and soil physicochemical properties (Gan et al., 2021). Co-occurrence network analysis revealed that most microbial genera were significantly correlated with C/N, TN, and AP (Figures 5C,D). Furthermore, soil carbon and nutrient transformations are related to the composition of soil and rhizosphere microbial communities, especially following the long-term application of biogas slurry (Czaban et al., 2018; Li Z. et al., 2023). *Nitrospira* was found to be significantly correlated with a variety of rhizosphere metabolites (Figure 6). This correlation may be attributed to the fact that plants mainly affect the diazotrophic bacteria through root metabolites (Lin et al., 2018). Research has indicated that organic acids and saccharides are the main drivers of shifts in rhizosphere soil microbial communities (Shi et al., 2011). Our findings showed that organic acids were significantly positively correlated with C/N as well as with bacterial genus *Nitrospira*, but negatively correlated with fungal genus *Mortierella*. Organic acids supply substantial nutrients, particularly carbon sources, and thus exhibit a positive link with the flora in the soil that responds to the corresponding nutrients, In contrast, there is a negative correlation between organic acids and *Haliangium*, which is a predatory bacterium involved in environmental remediation processes (Zhang et al., 2023). Biogas slurry contributes a considerable amount of readily available phosphorus nutrients, enhancing the plant’s capacity to fix carbon dioxide into sugars through photosynthesis. This results in a significant positive correlation between saccharides and AP. However, the genus *Flavobacterium* possesses a wide range of polysaccharide-degrading enzymes involved in sugar metabolism (Gavriilidou et al., 2020), therefore, it is negatively correlated with saccharides (Figure 5C).

Esters showed a positive correlation with TN and a negative correlation with *Flavobacterium* and *Geobacter*, genera known for their potential in heavy metal remediation. In addition, nucleosides and nucleotides exhibited a negative correlation with *Plectosphaerella*, a common phytopathogen, and a positive correlation with *Mortierella*, which has a beneficial biocontrol effect (Figure 5D). Previous studies have emphasized that plant root metabolites influence rhizosphere microorganisms, including the inhibition of pathogens (Hu et al., 2018; Zhou et al., 2023). Our findings disclosed a significant increase in the relative abundance of *Plectosphaerella* and *Mortierella*, suggesting that use of biogas slurry may potentially elevate the risk of certain pathogens, but also promote the growth of beneficial microorganisms (Huang et al., 2022). Heatmap analysis further indicated that *Plectosphaerella* was significantly correlated with metabolites related to both carbohydrate and fatty acid metabolism, whereas *Mortierella* and *Trichoderma* were significantly positively correlated with nucleic acid metabolism-related metabolites (Figure 6C). We propose that nucleosides and nucleotides may contribute to enhancing disease resistance in both the plant’s internal and soil environments, which is consistent with previous findings (Alferez et al., 2018; Lu et al., 2023). Long-term application of biogas slurry markedly enriched key metabolic pathways related to fatty acid, carbohydrate, and nucleic acid metabolism (Figure 4). They showed significant correlations with rhizosphere microorganisms, with bacterial communities showing particularly strong responses (Figures 1C, 6). Under low-concentration biogas slurry, the decrease in organic acid metabolites accompanied by increased community alpha diversity suggests a microbial shift from r- to K-strategy as nutrients become abundant, enhancing substrate utilization efficiency and promoting organic acid assimilation and soil carbon sequestration (Liang et al., 2024). The elevated abundance of pyrimidine metabolites (uracil, cytosine) reflects accelerated microbial cell turnover and RNA synthesis, indicating an active proliferation state of rhizosphere microorganisms (Kempes et al., 2017). Moreover, enrichment of the galactose metabolism pathway contributed to soil aggregate stability by stimulating the synthesis of microbial extracellular polymeric substances (EPS) (Six et al., 2004). Overall, low-concentration biogas slurry application improved soil ecological health through the synergistic regulation of microorganisms and metabolites.

The effect of biogas slurry application on the rhizosphere soil system is concentration-dependent. As the concentration of biogas slurry increased, the microbial community structure changed significantly (Figure 7). This negative effect might be attributed to the increase in plant-pathogenic microorganisms, such as *Plectosphaerella*, in the nutrient-rich environment. Additionally, appropriate alterations in soil physicochemical properties actively promoted the microbial community structure and diversity, leading to more active the colonization and functional expression of beneficial microorganisms (He et al., 2025). The extremely significant negative effect of soil microbial structure on metabolites may stem from microbial utilization and consumption: the more “active” the microbial community, the more readily “extractable small-molecule metabolites” are converted into “microbial biomass carbon” or “gaseous carbon,” resulting in a highly significant negative path coefficient. In contrast, the positive influence of microbial community structure on diversity implies the enhanced functional redundancy and niche differentiation (Morgunov et al., 2017). Notably, although both soil properties and microbial community structure can directly influence rhizosphere metabolites, the latter plays the dominant role. In summary, the observed correlations among key microbial taxa, biogas slurry, root exudates, and soil properties highlight the functional complexity of the rhizosphere community. This complexity, as evidenced by the multidimensional interaction network, supports the functional stability of the ecosystem. The simultaneous presence of positive and negative associations may enhance the microbial community’s capacity to buffer environmental fluctuations, thereby helping to maintain the stability of core ecosystem functions under sustained inputs such as biogas slurry.

## 5 Conclusion

In this study, we analyzed the variances in physicochemical properties, microbial community structure, and metabolite profiles of poplar rhizosphere soils treated with different concentrations of biogas slurry to explore the comprehensive influences of slurry application on rhizosphere microdomains. We found that the application of biogas slurry enhanced soil fertility, and the relevant microbial communities involved in the soil nutrient cycle have a positive response. However, high-concentration biogas slurry poses risks of heavy metal pollution and pathogen invasion, indicated by changes in environmental indicator microbe abundance and increased pathogen levels. Untargeted metabolomics analysis revealed that the number of differential metabolites in the Low vs. Con comparison group was significantly greater. The application of biogas slurry promoted the pyrimidine metabolic process and inhibited fatty acid biosynthesis, resulting in a significant decrease in the content of organic acids, while significantly increasing the levels of nucleosides and nucleotides, saccharides, and esters. Co-occurrence network analysis revealed substantial correlations between rhizosphere soil metabolites and microbes, indicating a more complex bacterial-soil property-metabolite network compared to the fungal network. Moreover, nucleosides and nucleotides are closely related to the suppression of soil-borne pathogens and the recruitment of beneficial microorganisms. Most of the differential metabolites in the nucleotide metabolic pathway were significantly negatively correlated with bacterial genera such as *Pseudomonas*, *AKYG587*, and *Nitrospira*, and significantly positively correlated with fungal genera including *Mortierella*, *Trichoderma*, and *Ciboria*. Overall, applying low concentrations of biogas slurry had a greater effect on rhizosphere metabolites and recruited beneficial rhizosphere microorganisms, with a relatively low possibility of heavy metal contamination and plant diseases. These findings contribute to a better understanding of the response mechanisms within the microdomains of rhizosphere soil in poplar plantations subjected to biogas slurry application and provide valuable insights for scientific fertilization practices in forestry.

## Data Availability

The datasets presented in this study can be found in online repositories. The names of the repository/repositories and accession number(s) can be found in the article/Supplementary material.

## Footnotes

1
http://www.mothur.org

2
https://www.chiplot.online/
