# Supplementary material for: Long-term biogas slurry fertilization alters rhizosphere soil metabolite profiles and microbial communities in poplar plantations
Source: Front Microbiol. 2026 Jan 6;16:1727035. doi: 10.3389/fmicb.2025.1727035 (PMC12816282; doi:10.3389/fmicb.2025.1727035)
Supplement: Supplementary file 1 [file Data_Sheet_1.pdf]

Supplementary Material

Long-term biogas slurry fertilization alters rhizosphere soil metabolite profiles and microbial communities in poplar plantations

Xiao-Xiao Gao[1, †], Xing-Ye Yu[1, †], Chun-Zhi Jin[2], Long Jin [1], Su Bu [3], Taihua Li [1, 4], Hong-Hua Ruan [1], Kee-Sun Shin[2, *] and Feng-Jie Jin [1, *]

[1] College of Ecology and Environment, Co-Innovation Center for Sustainable Forestry in Southern China, Nanjing Forestry University, 159 Longpan Road, Nanjing 210037, China.

[2] Korea Research Institute of Bioscience and Biotechnology (KRIBB), Daejeon 34141, Republic of Korea

[3] College of Life Science, Nanjing Forestry University, Nanjing 210037, China

[4] National Positioning Observation Station of Hung-tse Lake Wetland Ecosystem in Jiangsu Province, Hongze 223100, China

Figure S1 Venn diagram analysis. The numbers of overlapped and unique OTUs of bacterial (A) and fungal (B) communities in poplar rhizosphere soil among groups are shown in the Venn diagram.

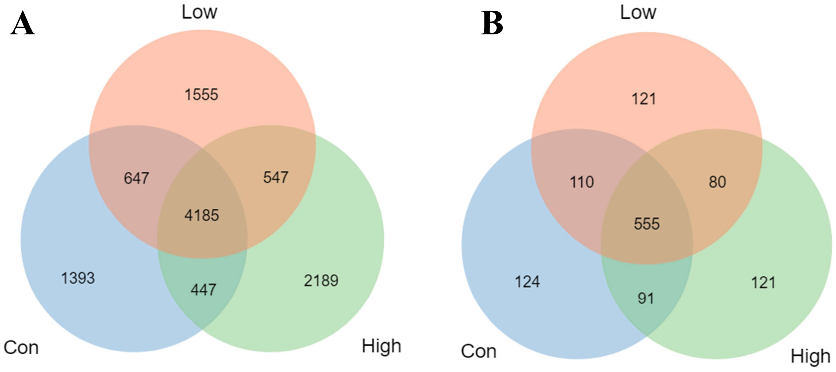

Figure S2. Volcanic maps of differential metabolites between different treatments. (C) Low vs. Con; (D) High vs. Con.

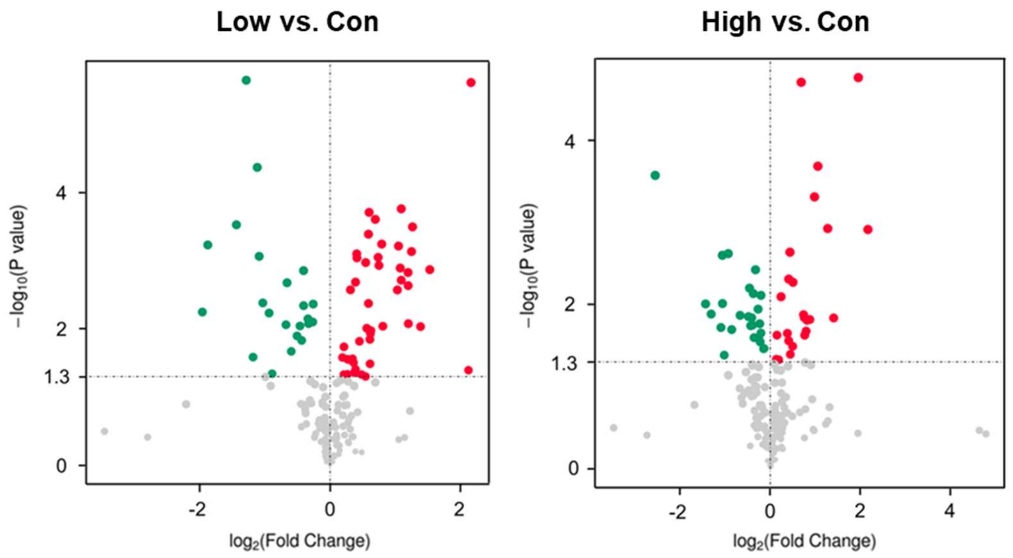

Figure S3. Cluster analysis of differential metabolites in rhizosphere soil treated with three concentrations of biogas slurry.

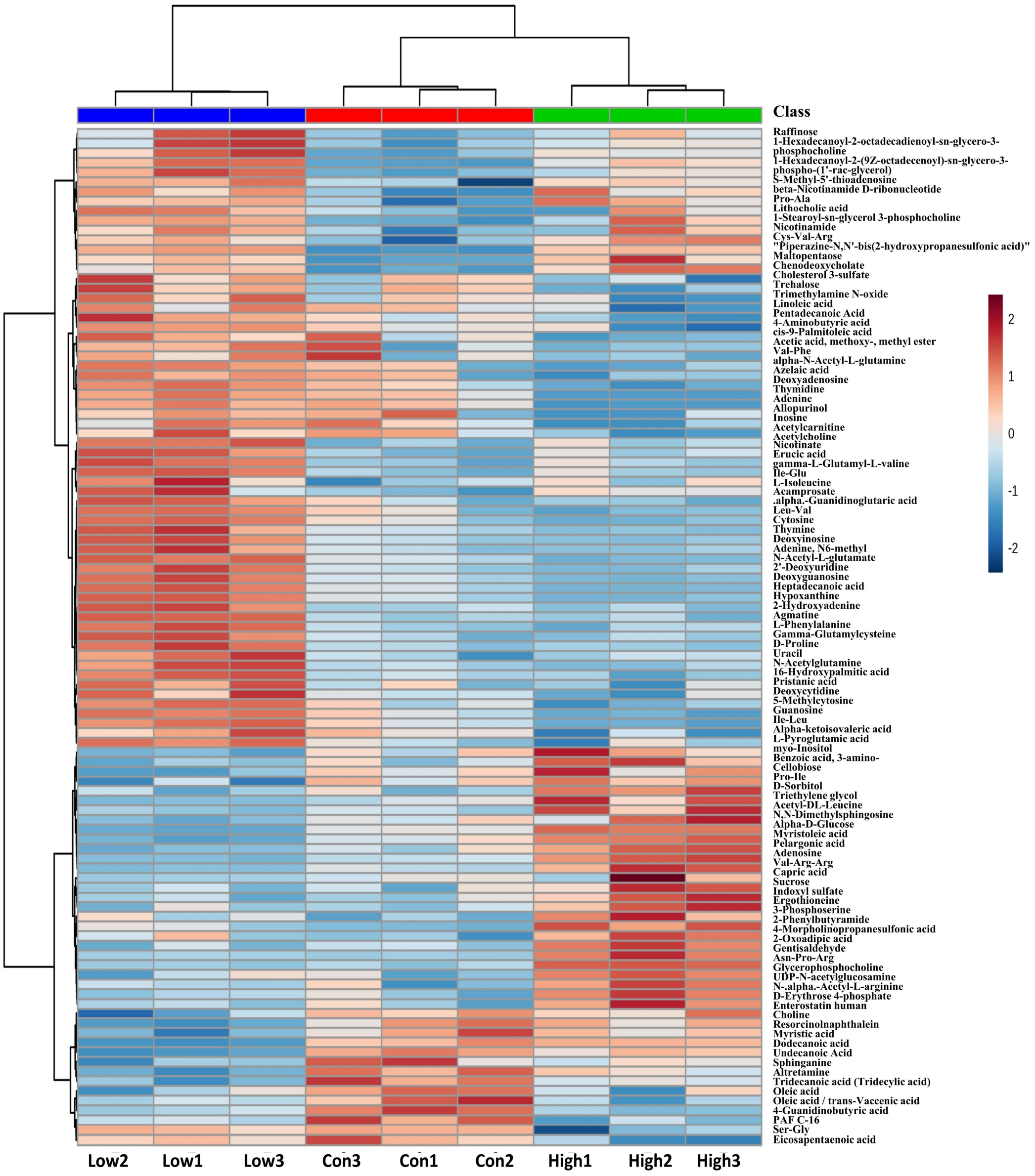

Table S1. The initial physicochemical properties of the soil in the experimental plots

Soil layer (cm)	pH	Moisture content (100%)	Volume weight of soil (g cm^{-3})	Total N (g kg^{-1})	Total P (g kg^{-1})	Nitrate (mg kg^{-1})	Ammonium (mg kg^{-1})
0-10	8.6	23.8	1.26	1.27	0.68	3.64	2.07

Table S2 Fundamental physicochemical characteristics of biogas liquid

pH	Total N (mg L^{-1})	Ammonium (mg L^{-1})	Nitrate (mgL^{-1})	Total P (mg L^{-1})	DOC (mg L^{-1})
8.15	667.96	438.13	2.25	69.62	501.1

Table S3. Relative abundance of the bacterial community composition in rhizospheric soil at the genus level (Top 30)

**Genus**	Con	Low	High
(Unassigned)	0.6211 ± 0.0012 b	0.6457 ± 0.0005 a	0.6144 ± 0.0005 c
uncultured	0.1404 ± 0.0016 a	0.1463 ± 0.0010 a	0.1393 ± 0.0013 a
RB41	0.0288 ± 0.0015 b	0.0318 ± 0.0008 b	0.0482 ± 0.0013 a
MND1	0.0242 ± 0.0008 a	0.0254 ± 0.0008 b	0.0243 ± 0.0004 a
Nitrospira	0.0249 ± 0.0002 a	0.0196 ± 0.0004 a	0.0243 ± 0.0004 a
Pseudomonas	0.0060 ± 0.0005 a	0.0041 ± 0.0001 b	0.0237 ± 0.0019 b
Flavobacterium	0.0152 ± 0.0017 a	0.0077 ± 0.0006 c	0.0054 ± 0.0003 b
Pseudolabrys	0.0082 ± 0.0002 a	0.0095 ± 0.0002 b	0.0103 ± 0.0004 b
Haliangium	0.0079 ± 0.0004 b	0.0091 ± 0.0001 a	0.0084 ± 0.0003 a
Geobacter	0.0140 ± 0.0004 b	0.0031 ± 0.0000 b	0.0058 ± 0.0000 a
AKYG587	0.0059 ± 0.0002 a	0.0057 ± 0.0002 a	0.0086 ± 0.0002 a
Massilia	0.0097 ± 0.0003 b	0.0044 ± 0.0001 b	0.0041 ± 0.0001 a
Dongia	0.0053 ± 0.0002 a	0.0057 ± 0.0001 b	0.0074 ± 0.0003 c
SWB02	0.0051 ± 0.0001 a	0.0055 ± 0.0003 a	0.0075 ± 0.0004 a
Bryobacter	0.0058 ± 0.0001 a	0.0060 ± 0.0001 a	0.0053 ± 0.0001 b
Subgroup_10	0.0067 ± 0.0004 b	0.0059 ± 0.0002 b	0.0037 ± 0.0001 a
Polycyclovorans	0.0031 ± 0.0001 a	0.0069 ± 0.0002 b	0.0038 ± 0.0001 b
PAUC26f	0.0031 ± 0.0001 a	0.0069 ± 0.0001 b	0.0038 ± 0.0003 b
Gaiella	0.0049 ± 0.0002 b	0.0037 ± 0.0002 b	0.0045 ± 0.0003 a
IS-44	0.0058 ± 0.0002 ab	0.0040 ± 0.0001 a	0.0025 ± 0.0001 b
Acidibacter	0.0029 ± 0.0002 a	0.0031 ± 0.0002 a	0.0046 ± 0.0001 a
mle1-7	0.0051 ± 0.0001 a	0.0024 ± 0.0001 a	0.0028 ± 0.0001 b
Candidatus_Solibacter	0.0032 ± 0.0002 a	0.0030 ± 0.0002 b	0.0036 ± 0.0001 b
Lysobacter	0.0055 ± 0.0002 b	0.0024 ± 0.0001 a	0.0014 ± 0.0001 b
Ralstonia	0.0031 ± 0.0005 a	0.0054 ± 0.0006 a	0.0005 ± 0.0001 a
Terrimonas	0.0026 ± 0.0001 a	0.0037 ± 0.0002 b	0.0031 ± 0.0001 b
Sphingomonas	0.0021 ± 0.0001 b	0.0030 ± 0.0000 a	0.0044 ± 0.0000 ab
Mesorhizobium	0.0020 ± 0.0000 a	0.0026 ± 0.0001 b	0.0031 ± 0.0001 a
Ellin6067	0.0035 ± 0.0003 a	0.0017 ± 0.0001 b	0.023 ± 0.0000 ab
SM1A02	0.0027 ± 0.0001 b	0.0020 ± 0.0000 b	0.0028 ± 0.0001 a

Values are Mean ± standard error (n=3); Different lowercase letters within the same row indicate significant differences ($P < 0.05$) among treatments. Con - no treatment control; Low - biogas slurry (250 m^3 ha^{-1} yr^{-1}) treatment; High - biogas slurry (375 m^3 ha^{-1} yr^{-1}) treatment.

Table S4. Relative abundance of the fungal community composition in rhizospheric soil at the genus level (Top 30)

**Genus**	Con	Low	High
(Unassigned)	0.4828± 0.0434 a	0.4797 ± 0.0187 a	0.5707 ± 0.0035 a
unidentified	0.0790 ± 0.0082 a	0.1126 ± 0.0053 a	0.0941 ± 0.0055 a
Geopora	0.1959 ± 0.0481 a	0.0039 ± 0.0010 b	0.0067 ± 0.0007 b
Mortierella	0.0168 ± 0.0037 b	0.1784 ± 0.0414 a	0.0329 ± 0.0063 b
Plectosphaerella	0.0014 ± 0.0001 b	0.0011 ± 0.0000 b	0.1211 ± 0.0287 a
Humicola	0.0257 ± 0.0024 a	0.0328 ± 0.0027 a	0.0293 ± 0.0059 a
Fusarium	0.0274 ± 0.0046 a	0.0244 ± 0.0028 a	0.0222 ± 0.0008 a
Sarocladium	0.0574 ± 0.0320 a	0.0008 ± 0.0001 a	0.0009 ± 0.0001 a
Cladorrhinum	0.0046 ± 0.0007 a	0.0039 ± 0.0007 a	0.0422 ± 0.0195 a
Staphylotrichum	0.0165 ± 0.0027 a	0.0176 ± 0.0041 a	0.0100 ± 0.0005 a
Ascobolus	0.0077 ± 0.0038 a	0.0229 ± 0.0099 a	0.0048 ± 0.0007 a
Helicotylenchus	0.0003 ± 0.0001 a	0.0270 ± 0.0135 a	0.0030 ± 0.0011 a
Ciboria	0.0009 ± 0.0001b	0.0299 ± 0.0031 a	0.0031 ± 0.0012 b
Trichoderma	0.0071 ± 0.0006 ab	0.0111 ± 0.0014 a	0.0031 ± 0.0012 b
Pyrenochaeta	0.0138 ± 0.0027 a	0.0036 ± 0.0010 b	0.0018 ± 0.0002 b
Clonostachys	0.0065 ± 0.00105 a	0.0053 ± 0.0022 a	0.0039 ± 0.0012 a
Tylenchorhynchus	0.0001 ± 0.0000 a	0.0003 ± 0.0001 a	0.0128 ± 0.0073 a
Tomentella	0.0119 ± 0.0056 a	0.0005 ± 0.0000 a	0.0005 ± 0.0001 a
Aspergillus	0.0071 ± 0.0023 a	0.0030 ± 0.0006 a	0.0025 ± 0.0001 a
Tetracladium	0.0048 ± 0.0007 a	0.0029 ± 0.0006 a	0.0017 ± 0.0004 a
Ramophialophora	0.0030 ± 0.0004 a	0.0034 ± 0.0003 a	0.0017 ± 0.0001 a
Roussoella	0.0030 ± 0.00004 a	0.0034 ± 0.0003 a	0.0017 ± 0.0001 a
Striatibotrys	0.0012 ± 0.0002 a	0.0021 ± 0.0005 a	0.0031 ± 0.0011a
Verticillium	0.0003 ± 0.0000 a	0.0004 ± 0.0001 a	0.0050 ± 0.0020 a
Prorodon	0.0002 ± 0.0000 a	0.0028 ± 0.0015 a	0.0023 ± 0.0013 a
Inocybe	0.0014 ± 0.0001 a	0.0020 ± 0.0001 a	0.0016 ± 0.0002 a
Glomus	0.0010 ± 0.0002 b	0.0032 ± 0.0013 b	0.0003 ± 0.0000 a
Paracremonium	0.0018 ± 0.0000 a	0.0006 ± 0.0003 b	0.0007 ± 0.0002 b
Cyrtohymena	0.0014 ± 0.0006 b	0.0011 ± 0.0001 ab	0.0008 ± 0.0002 a
Beauveria	0.0007 ± 0.0003 a	0.0015 ± 0.006 a	0.0007 ± 0.0002 a

Values are Mean ± standard error (n=3); Different lowercase letters within the same row indicate significant differences (*P* < 0.05) among treatments. Con - no treatment control; Low - biogas slurry (250 m^3 ha^{-1} yr^{-1}) treatment; High - biogas slurry (375 m^3 ha^{-1} yr^{-1}) treatment.

Table S5. Relative contents of metabolites in rhizosphere soil treated with three concentrations of biogas slurry

Metabolite category	Con	Low	High
Organic acids	0.7518 ± 0.0079 a	0.7088 ± 0.0030 b	0.7429 ± 0.0063 a
Nucleosides and nucleotides	0.0718 ± 0.0059 b	0.0910 ± 0.0006 a	0.0574 ± 0.0022 b
Organic bases	0.0731 ± 0.0031 a	0.0763 ± 0.0021 a	0.0736 ± 0.0006 a
Saccharides	0.0471 ± 0.0002 b	0.0562 ± 0.0010 a	0.0610 ± 0.0022 a
Esters	0.0171 ± 0.0005 b	0.0271 ± 0.0010 a	0.0253 ± 0.0013 a
Amino acids	0.0173 ± 0.0006 a	0.0188 ± 0.0004 a	0.0169 ± 0.0008 a
Alcohols	0.0113 ± 0.0006 a	0.0103 ± 0.0002 a	0.0114 ± 0.0000 a
Others	0.0107 ± 0.0003 a	0.0117 ± 0.0002 a	0.0116 ± 0.0006 a

Values are Mean ± standard error (n=3); Different lowercase letters within the same row indicate significant differences ($P < 0.05$) among treatments. Con - no treatment control; Low - biogas slurry (250 m^3 ha^{-1} yr^{-1}) treatment; High - biogas slurry (375 m^3 ha^{-1} yr^{-1}) treatment.

Table S6. Analysis of significant differential metabolites in rhizosphere soil between different treatments (Low vs. Con).

**Metabolite_Name**	**Mean-Con**	**Mean-Low**	***P*-value**	**Log2FC**	**VIP**	**Class**
Resorcinolnaphthalein	0.1591	0.041	0.00563	-1.9543	1.33617	Others
4-Guanidinobutyric acid	10.7354	2.9351	0.00058	-1.8709	1.38867	Organic acids
Dodecanoic acid	62.5896	23.1849	0.00029	-1.4327	1.41794	Organic acids
Myristoleic acid	11.6602	4.7924	0	-1.2828	1.43266	Organic acids
"Benzoic acid, 3-amino-"	5.3206	2.3501	0.02586	-1.1789	1.27768	Organic acids
Undecanoic Acid	3.4135	1.5768	0.00004	-1.1142	1.41693	Organic acids
Val-Arg-Arg	0.0485	0.0229	0.00086	-1.0851	1.4056	Amino acids
Sucrose	0.1347	0.066	0.00414	-1.0295	1.34424	Saccharides
Pro-Ile	0.7358	0.3854	0.00583	-0.93321	1.35519	Amino acids
D-Sorbitol	0.7825	0.4232	0.04532	-0.88675	1.19767	Saccharides
Acetyl-DL-Leucine	0.0808	0.0506	0.00865	-0.6748	1.31424	Amino acids
Pelargonic acid	18.6841	11.8372	0.00209	-0.65848	1.39363	Organic acids
Sphinganine	1.4257	0.9445	0.02131	-0.59407	1.23449	Amino acids
Choline	6.0249	4.2438	0.01263	-0.50558	1.29614	Organic bases
Oleic acid	1.0762	0.782	0.00903	-0.46061	1.29883	Organic acids
Capric acid	19.4061	14.3575	0.0147	-0.43471	1.30051	Organic acids
Altretamine	0.1004	0.0758	0.00139	-0.40584	1.40664	Others
Tridecanoic acid (Tridecylic acid)	5.0135	3.786	0.00453	-0.40515	1.37494	Organic acids
PAF C-16	1.1124	0.8797	0.00709	-0.33852	1.36003	Organic bases
Myristic acid	26.8025	21.3354	0.00833	-0.32912	1.33057	Organic acids
Alpha-D-Glucose	1.3114	1.0906	0.00789	-0.26601	1.35278	Saccharides
Oleic acid / trans-Vaccenic acid	94.7607	79.2026	0.0043	-0.25874	1.35195	Organic acids
Nicotinamide	0.4241	0.4835	0.0262	0.18909	1.22201	Others
Raffinose	2.6169	3.0262	0.04666	0.20968	1.17922	Saccharides
myo-Inositol	0.5281	0.6118	0.01827	0.21237	1.26549	Alcohols
Acamprosate	7.4587	8.9699	0.04615	0.26616	1.16666	Organic acids
Ile-Leu	0.2293	0.276	0.02776	0.26723	1.22261	Amino acids
"Piperazine-N,N'-bis(2-hydroxypropanesulfonic acid)"	6.373	7.7294	0.02818	0.27837	1.20951	Organic acids
Heptadecanoic acid	42.1512	52.302	0.00265	0.31129	1.37279	Organic acids
Guanosine	0.1289	0.1636	0.02733	0.34382	1.20747	Nucleosides and nucleotides
Maltopentaose	0.7267	0.9327	0.03127	0.36006	1.18283	Saccharides
Leu-Val	0.1851	0.2391	0.04378	0.36917	1.16895	Amino acids
16-Hydroxypalmitic acid	6.9064	9.046	0.00205	0.38935	1.39404	Organic acids
5-Methylcytosine	0.0162	0.0213	0.03916	0.39002	1.18844	Nucleosides and nucleotides
Trehalose	31.3142	41.5528	0.00079	0.40813	1.413	Saccharides

S-Methyl-5'-thioadenosine	3.2926	4.3775	0.0009	0.41088	1.40332	Nucleosides and nucleotides
1-Stearoyl-sn-glycerol 3-phosphocholine	0.0306	0.0418	0.01516	0.44947	1.26348	Organic bases
Cytosine	1.9466	2.7182	0.04665	0.48172	1.17663	Nucleosides and nucleotides
1-Hexadecanoyl-2-octadecadienoyl -sn-glycero-3-phosphocholine	3.8506	5.6085	0.04974	0.54251	1.16984	Organic bases
Uracil	0.3438	0.5015	0.00106	0.54454	1.39855	Nucleosides and nucleotides
Lithocholic acid	0.4937	0.7286	0.00974	0.56144	1.30012	Organic acids
Pristanic acid	1.9023	2.8576	0.0004	0.58709	1.4225	Organic acids
D-Proline	0.6477	0.9733	0.00421	0.58756	1.35342	Amino acids
L-Phenylalanine	0.1974	0.2987	0.00019	0.59773	1.40481	Amino acids
Thymine	2.9039	4.4288	0.0142	0.6089	1.29515	Nucleosides and nucleotides
Deoxycytidine	0.2782	0.4249	0.03246	0.61102	1.24518	Nucleosides and nucleotides
N-Acetyl-L-glutamate	0.1552	0.2381	0.01166	0.61715	1.29877	Esters
Alpha-ketoisovaleric acid	2.6446	4.0751	0.01055	0.62382	1.31104	Organic acids
Cholesterol 3-sulfate	7.2369	11.6909	0.00024	0.69194	1.41643	Esters
L-Isoleucine	0.8183	1.3621	0.00089	0.73512	1.39196	Amino acids
Agmatine	0.0593	0.0995	0.00116	0.74763	1.38119	Others
Gamma-Glutamylcysteine	0.4046	0.6996	0.00056	0.79007	1.40285	Amino acids
1-Hexadecanoyl-2-(9Z-octadecenoyl) -sn-glycero-3-phospho-(1'-rac-glycerol)	7.5263	13.1692	0.00909	0.80714	1.32819	Esters
"Adenine, N6-methyl"	0.2263	0.462	0.00266	1.0297	1.36884	Nucleosides and nucleotides
Ile-Glu	0.6237	1.2884	0.0006	1.0468	1.40257	Amino acids
2-Hydroxyadenine	1.7944	3.776	0.00127	1.0734	1.3908	Nucleosides and nucleotides
gamma-L-Glutamyl-L-valine	0.2596	0.5521	0.00192	1.0888	1.36985	Amino acids
Cys-Val-Arg	0.0387	0.0824	0.00017	1.0891	1.41874	Amino acids
Hypoxanthine	1.004	2.295	0.00148	1.1927	1.39044	Nucleosides and nucleotides
Deoxyguanosine	1.0962	2.5081	0.0023	1.1942	1.38102	Nucleosides and nucleotides
N-Acetylglutamine	0.1269	0.2911	0.00834	1.1974	1.3394	Others
2'-Deoxyuridine	3.1878	7.5601	0.00073	1.2458	1.40212	Nucleosides and nucleotides
Erucic acid	0.7795	1.8679	0.00032	1.2609	1.42548	Organic acids
Deoxyinosine	0.0464	0.1212	0.00921	1.3838	1.31213	Nucleosides and nucleotides
beta-Nicotinamide D-ribonucleotide	0.085	0.2449	0.00134	1.5257	1.39034	Nucleosides and nucleotides

.alpha.-Guanidinoglutaric acid	0.2273	0.986	0.04029	2.1171	1.17642	Organic acids
Chenodeoxycholate	0.2387	1.0639	0	2.1563	1.43454	Others

Table S7 Analysis of significant differential metabolites in rhizosphere soil between different treatments (High vs. Con).

Metabolite Name	Mean-Con	Mean-High	*P*-value	Log2FC	VIP	Class
4-Guanidinobutyric acid	10.7354	1.8164	0.00027	-2.5632	1.47857	Organic acids
2'-Deoxyuridine	3.1878	1.1737	0.00984	-1.4415	1.39346	Nucleosides and nucleotides
Hypoxanthine	1.0040	0.4030	0.01307	-1.3169	1.37540	Nucleosides and nucleotides
Deoxyinosine	0.0464	0.0217	0.01906	-1.0992	1.34623	Nucleosides and nucleotides
Allopurinol	0.8238	0.3930	0.00252	-1.0678	1.44918	Others
Alpha-ketoisovaleric acid	2.6446	1.2639	0.00974	-1.0651	1.38844	Organic acids
L-Pyroglutamic acid	2.4156	1.1865	0.04141	-1.0256	1.25407	Amino acids
Inosine	0.5673	0.2964	0.00240	-0.93653	1.45001	Nucleosides and nucleotides
2-Hydroxyadenine	1.7944	0.9887	0.02021	-0.85997	1.34165	Nucleosides and nucleotides
Eicosapentaenoic acid	4.4678	2.8035	0.01355	-0.67231	1.35981	Organic acids
Adenine	4.4748	3.1933	0.01406	-0.48679	1.36775	Nucleosides and nucleotides
PAF C-16	1.1124	0.8074	0.00632	-0.46228	1.40174	Organic bases
Oleic acid	1.0762	0.7949	0.01814	-0.43708	1.32721	Organic acids
Nicotinate	0.8020	0.6018	0.01451	-0.41429	1.36239	Others
Ile-Leu	0.2293	0.1740	0.01786	-0.39793	1.34694	Amino acids
Ser-Gly	0.2972	0.2286	0.00736	-0.3782	1.39993	Amino acids
Acetylcholine	1.0592	0.8248	0.02533	-0.36087	1.31669	Organic bases
Oleic acid / trans-Vaccenic acid	94.7607	75.4321	0.00377	-0.32911	1.41139	Organic acids
Linoleic acid	0.4777	0.3922	0.02559	-0.28427	1.29082	Organic acids
Tridecanoic acid (Tridecylic acid)	5.0135	4.1524	0.01140	-0.27186	1.36918	Organic acids
Undecanoic Acid	3.4135	2.8623	0.02641	-0.25409	1.30691	Organic acids
cis-9-Palmitoleic acid	119.7417	101.7130	0.01715	-0.23542	1.32989	Organic acids
4-Aminobutyric acid	0.1550	0.1327	0.02863	-0.22431	1.29128	Organic acids
Altretamine	0.1004	0.0869	0.02227	-0.20871	1.30301	Others
Heptadecanoic acid	42.1512	36.4824	0.00774	-0.20837	1.40552	Organic acids
Pentadecanoic Acid	81.7192	73.6382	0.03461	-0.15022	1.25518	Organic acids
16-Hydroxypalmitic acid	6.9064	6.2706	0.03436	-0.13933	1.28698	Organic acids
2-Phenylbutyramide	0.7118	0.7811	0.04647	0.13417	1.22003	Others
2-Oxoadipic acid	13.2327	14.6686	0.02363	0.14863	1.28875	Organic acids
Triethylene glycol	0.9076	1.0327	0.04705	0.18628	1.21842	Alcohols
S-Methyl-5'-thioadenosine	3.2926	3.8873	0.00805	0.23954	1.37896	Nucleosides and nucleotides
Adenosine	8.3067	10.8141	0.02248	0.38057	1.31921	Nucleosides and nucleotides
Maltopentaose	0.7267	0.9659	0.02768	0.41055	1.29328	Saccharides
1-Hexadecanoyl-2-(9Z-octadecenoyl)-sn-glycero-3-phospho-(1'-rac-glycerol)	7.5263	10.0062	0.00490	0.41088	1.42624	Esters
Pelargonic acid	18.6841	25.3042	0.00231	0.43757	1.44894	Organic acids
1-Stearoyl-sn-glycerol 3-phosphocholine	0.0306	0.0417	0.04051	0.44551	1.24774	Organic bases
Lithocholic acid	0.4937	0.6972	0.03231	0.49778	1.27389	Organic acids
Trehalose	31.3142	44.4335	0.00536	0.50483	1.40124	Saccharides
Myristoleic acid	11.6602	18.7878	0.00002	0.6882	1.49424	Organic acids
Capric acid	19.4061	32.4142	0.01333	0.74011	1.34431	Organic acids
UDP-N-acetylglucosamine	0.0649	0.1097	0.01443	0.75627	1.34344	Others
"N,N-Dimethylsphingosine"	0.4137	0.7032	0.02367	0.76554	1.30144	Alcohols
Cholesterol 3-sulfate	7.2369	12.5771	0.02110	0.79736	1.31607	Esters
beta-Nicotinamide D-ribonucleotide	0.0850	0.1501	0.01555	0.81937	1.34487	Nucleosides and nucleotides

3-Phosphoserine	0.4782	0.8797	0.01524	0.87923	1.34559	Organic acids
4-Morpholinopropanesulfonic acid	0.1746	0.3453	0.00049	0.98423	1.47189	Organic acids
Glycerophosphocholine	0.8606	1.7938	0.00021	1.0596	1.48353	Organic bases
Val-Arg-Arg	0.0485	0.1177	0.00119	1.2789	1.45560	Amino acids
.alpha.-Guanidinoglutaric acid	0.2273	0.6044	0.01459	1.4111	1.33582	Organic acids
Chenodeoxycholate	0.2387	0.9290	0.00002	1.9607	1.49209	Others
Asn-Pro-Arg	0.0069	0.0311	0.00122	2.1745	1.45326	Amino acids

Mean, Mean relative quantitative value; VIP, Variable Importance in Projection (OPLS-DA); Log2FC, Log2 Fold Change

Table S8 KEGG pathway enrichment analysis of differential metabolites in rhizosphere soil between treatments (Top 20).

Group	KEGG Pathway	Total	Hits	*P*-value	Impact	cpd_hits
Low vs. Con	Valine, leucine and isoleucine degradation	42	2	0.2607	0.04761	cpd:C00141 Alpha-ketoisovaleric acid;cpd:C00407 L-Isoleucine;
	Valine, leucine and isoleucine biosynthesis	23	2	0.1006	0.08695	cpd:C00407 L-Isoleucine;cpd:C00141 Alpha-ketoisovaleric acid;
	Steroid hormone biosynthesis	99	1	0.9126	0.01010	cpd:C18043 Cholesterol 3-sulfate;
	Secondary bile acid biosynthesis	35	2	0.1989	0.05714	cpd:C03990 Lithocholic acid;cpd:C02528 Chenodeoxycholate;
	Pyrimidine metabolism	68	5	0.01972	0.07352	cpd:C00106 Uracil;cpd:C00178 Thymine;cpd:C02376 5-Methylcytosine;cpd:C00380 Cytosine;cpd:C00881 Deoxycytidine;
	Purine metabolism	92	4	0.1681	0.04347	cpd:C05512 Deoxyinosine;cpd:C00262 Hypoxanthine;cpd:C00330 Deoxyguanosine;cpd:C00387 Guanosine;
	Primary bile acid biosynthesis	47	1	0.6796	0.02127	cpd:C02528 Chenodeoxycholate;
	Phenylalanine, tyrosine and tryptophan biosynthesis	35	1	0.5702	0.02857	cpd:C00079 L-Phenylalanine;
	Phenylalanine metabolism	72	1	0.8275	0.01388	cpd:C00079 L-Phenylalanine;
	Glycolysis / Gluconeogenesis	31	1	0.5262	0.03225	cpd:C00267 Alpha-D-Glucose;
	Glycine, serine and threonine metabolism	50	1	0.7024	0.02	cpd:C00114 Choline;
	Galactose metabolism	45	5	0.0034	0.11111	cpd:C00137 myo-Inositol;cpd:C00089 Sucrose;cpd:C00492 Raffinose;cpd:C00794 D-Sorbitol;cpd:C00267 Alpha-D-Glucose;
	Fructose and mannose metabolism	54	2	0.3666	0.03703	cpd:C00267 Alpha-D-Glucose;cpd:C00794 D-Sorbitol;
	Fatty acid biosynthesis	50	4	0.02794	0.08	cpd:C00712 Oleic acid;cpd:C06424 Myristic acid;cpd:C02679 Dodecanoic acid;cpd:C01571 Capric acid;
	Cyanoamino acid metabolism	45	2	0.2874	0.04444	cpd:C00407 L-Isoleucine;cpd:C00079 L-Phenylalanine;
	Cutin, suberine and wax biosynthesis	27	1	0.4779	0.03703	cpd:C00712 Oleic acid;
	beta-Alanine metabolism	32	1	0.5376	0.03125	cpd:C00106 Uracil;
	Ascorbate and aldarate metabolism	47	1	0.6796	0.02127	cpd:C00137 myo-Inositol;
	Arginine biosynthesis	23	1	0.4247	0.04347	cpd:C00624 N-Acetyl-L-glutamate;
	Arginine and proline metabolism	76	3	0.2653	0.03947	cpd:C01035 4-Guanidinobutyric acid;cpd:C00763 D-Proline;cpd:C00179 Agmatine;

	Valine, leucine and isoleucine degradation	42	1	0.529	0.02380	cpd:C00141 Alpha-ketoisovaleric acid;
	Valine, leucine and isoleucine biosynthesis	23	1	0.3362	0.04347	cpd:C00141 Alpha-ketoisovaleric acid;
	Tryptophan metabolism	81	1	0.77	0.01234	cpd:C00322 2-Oxoadipic acid;
	Steroid hormone biosynthesis	99	1	0.8358	0.0101	cpd:C18043 Cholesterol 3-sulfate;
	Starch and sucrose metabolism	51	1	0.6002	0.01960	cpd:C01083 Trehalose;
	Secondary bile acid biosynthesis	35	2	0.1239	0.05714	cpd:C03990 Lithocholic acid;cpd:C02528 Chenodeoxycholate;
	Purine metabolism	92	5	0.0196	0.05434	cpd:C05512Deoxyinosine;cpd:C00212 Adenosine;cpd:C00147 Adenine;cpd:C00294 Inosine;cpd:C00262 Hypoxanthine;
	Primary bile acid biosynthesis	47	1	0.5699	0.02127	cpd:C02528 Chenodeoxycholate;
High vs. Con	Neomycin, kanamycin and gentamicin biosynthesis	33	1	0.4456	0.03030	cpd:C00043 UDP-N-acetylglucosamine;
	Lysine degradation	52	1	0.6074	0.01923	cpd:C00322 2-Oxoadipic acid;
	Lysine biosynthesis	35	1	0.4653	0.02857	cpd:C00322 2-Oxoadipic acid;
	Linoleic acid metabolism	28	1	0.3933	0.035714	cpd:C01595 Linoleic acid;
	Glycine, serine and threonine metabolism	50	1	0.5928	0.02	cpd:C01005 3-Phosphoserine;
	Fatty acid biosynthesis	50	3	0.05477	0.06	cpd:C00712 Oleic acid;cpd:C08362 cis-9-Palmitoleic acid;cpd:C01571 Capric acid;
	Ether lipid metabolism	25	1	0.3597	0.04	cpd:C00670 Glycerophosphocholine;
	Cysteine and methionine metabolism	62	1	0.6732	0.01612	cpd:C01005 3-Phosphoserine;
	Cutin, suberine and wax biosynthesis	27	1	0.3823	0.03703	cpd:C00712 Oleic acid;
	beta-Alanine metabolism	32	1	0.4355	0.03125	cpd:C00334 4-Aminobutyric acid;
	Arginine and proline metabolism	76	2	0.3886	0.02631	cpd:C00334 4-Aminobutyric acid;cpd:C01035 4-Guanidinobutyric acid;
	Alanine, aspartate and glutamate metabolism	28	1	0.3933	0.03571	cpd:C00334 4-Aminobutyric acid;

Total: Total metabolites in the pathway; Hits: Differential metabolites mapped; Impact: Pathway importance score; cpd_hits: Mapped compound names.
